# Depletion of Arabidopsis ACYL-COA-BINDING PROTEIN3 Affects Fatty Acid Composition in the Phloem

**DOI:** 10.3389/fpls.2018.00002

**Published:** 2018-01-25

**Authors:** Tai-Hua Hu, Shiu-Cheung Lung, Zi-Wei Ye, Mee-Len Chye

**Affiliations:** School of Biological Sciences, The University of Hong Kong, Pokfulam, Hong Kong

**Keywords:** acyl-CoA esters, fatty acids, linolenic acid, jasmonate, oxylipins, wounding

## Abstract

Oxylipins are crucial components in plant wound responses that are mobilised via the plant vasculature. Previous studies have shown that the overexpression of an Arabidopsis acyl-CoA-binding protein, AtACBP3, led to an accumulation of oxylipin-containing galactolipids, and *AtACBP3pro::BETA-GLUCURONIDASE* (*GUS*) was expressed in the phloem of transgenic Arabidopsis. To investigate the role of AtACBP3 in the phloem, reverse transcription-polymerase chain reaction and western blot analysis of phloem exudates from the *acbp3* mutant and wild type revealed that the AtACBP3 protein, but not its mRNA, was detected in the phloem sap. Furthermore, micrografting demonstrated that AtACBP3 expressed from the *35S* promoter was translocated from shoot to root. Subsequently, AtACBP3 was localised to the companion cells, sieve elements and the apoplastic space of phloem tissue by immunogold electron microscopy using anti-AtACBP3 antibodies. *AtACBP3pro::GUS* was induced locally in Arabidopsis leaves upon wounding, and the expression of wound-responsive jasmonic acid marker genes (*JASMONATE ZIM-DOMAIN10, VEGETATIVE STORAGE PROTEIN2*, and *LIPOXYGENASE2*) increased more significantly in both locally wounded and systemic leaves of the wild type in comparison to *acbp3* and *AtACBP3-*RNAi. Oxylipin-related fatty acid (FA) (C18:2-FA, C18:3-FA and methyl jasmonate) content was observed to be lower in *acbp3* and *AtACBP3-*RNAi than wild-type phloem exudates using gas chromatography-mass spectrometry. Experiments using recombinant AtACBP3 in isothermal titration calorimetry analysis showed that medium- and long-chain acyl-CoA esters bind (His)_6_-AtACBP3 with *K_D_* values in the micromolar range. Taken together, these results suggest that AtACBP3 is likely to be a phloem-mobile protein that affects the FA pool and jasmonate content in the phloem, possibly by its binding to acyl-CoA esters.

## Introduction

To enhance survival, land plants have developed a complex mechanism to cope with wounding. Early evidence showed that mechanical wounding induces defence-related proteinase inhibitors and their transcripts from potato (*Solanum tuberosum*) and tomato (*S. lycopersicum*) (Graham et al., 1986; Farmer and Ryan, 1992) and the vasculature plays an important role in signal transduction for plant defence (for a review see Shah, 2009). The vasculature consists of the phloem and the xylem. Water and mineral nutrients are channelled to the source tissues of plants through the xylem (for a review see De Boer and Volkov, 2003), while the phloem, consisting of the parenchyma cells, sieve elements (SE) and companion cells (CC) (for a review see Schulz, 1998), delivers sucrose (Riesmeier et al., 1994), phytohormones (e.g., abscisic acid, auxins, cytokinins, and gibberellins) (for a review see Hoad, 1995), ribonucleic acids (Xoconostle-Cazares et al., 1999), proteins (Hayashi et al., 2000; Yoo et al., 2002; Carella et al., 2016) and lipids (Madey et al., 2002; Guelette et al., 2012). Putative lipid-transfer proteins (LTPs), such as *Arabidopsis thaliana* DEFECTIVE IN INDUCED RESISTANCE1 (AtDIR1), tomato DIR1 and a putative rice acyl-CoA-binding protein (ACBP), have been identified in the phloem and likely participate in lipid trafficking (Maldonado et al., 2002; Suzui et al., 2006; Mitton et al., 2009). Along with a recent proteomic study that revealed yet more LTPs with putative roles in lipid-mediated signalling in the phloem (Barbaglia et al., 2016), the vasculature, particularly the phloem, shows great potentials for plant scientists to study the mechanisms of lipid transport in plant defence.

Fatty acids (FAs) are an important source of energy reserve in all organisms and constitute the primary composition of lipids (for a review see Ohlrogge and Browse, 1995). In plants, FAs are also essential for the biosynthesis of jasmonic acid (JA) and its derivatives (together referred to jasmonate hereafter) which play many roles in defence (Creelman and Mullet, 1997; Vijayan et al., 1998; Sanders et al., 2000; Franke et al., 2005). Arabidopsides are galactolipid derivatives containing an oxygenated jasmonic precursor, 12-oxo-phytodienoic acid (OPDA) (Stelmach et al., 2001; Hisamatsu et al., 2003, 2005; Andersson et al., 2006; Buseman et al., 2006; Kourtchenko et al., 2007), and many of them have been directly associated with the plant wound responses (Buseman et al., 2006; Kourtchenko et al., 2007). Upon wounding, JA production has been reported to increase in locally wounded and systemic leaves suggesting transport of a systemic wound signal (Glauser et al., 2008, 2009; Mousavi et al., 2013). Glauser et al. (2009) also demonstrated that direct vascular connexions to wounded leaves are crucial to JA augmentation in systemic tissues.

In plants, FAs are activated by acyl-CoA synthetase and converted into acyl-CoA esters (Gerbling et al., 1994). ACBPs have been suggested to maintain the acyl-CoA pools in plants (Yurchenko et al., 2009). In Arabidopsis, six AtACBPs have been postulated to play different roles in phospholipid metabolism and membrane biogenesis as well as homeostasis (for reviews see Xiao and Chye, 2009, 2011a; Du et al., 2016; Ye and Chye, 2016). In Arabidopsis rosettes, the promoter activities of *AtACBP1*, *AtACBP3*, and *AtACBP6* are detectable in the phloem besides other tissues (Zheng et al., 2012; Xue et al., 2014; Ye et al., 2016a). AtACBP6 is involved in systemic acquired resistance (SAR) (Xia et al., 2012) and it accumulates in the phloem sap collected from SAR-induced leaves (Carella et al., 2016). Only AtACBP1 and AtACBP6 are known to be induced upon abiotic stress treatments (Chen et al., 2008; Xiao et al., 2008; Du et al., 2010, 2013; Liao et al., 2014). AtACBP1 and AtACBP6 also play overlapping roles with other AtACBPs in plant reproduction (Hsiao et al., 2015; Lung et al., 2017). Recently, AtACBP6 was found to be wound-inducible and to affect jasmonate composition in the phloem (Ye et al., 2016a). So far, only the overexpression of *AtACBP3* conferred protection against *Pseudomonas syringae* DC3000 in transgenic Arabidopsis besides inducing premature leaf senescence (Xiao et al., 2010; Xiao and Chye, 2011b). These phenotypes are likely caused by a shift in lipid homeostasis in the *AtACBP3-*overexpressors (*AtACBP3*-OEs) because they accumulated higher amounts of C18:3-CoA esters, phosphatidylethanolamine, lysophospholipids and arabidopsides with a significant decrease in phosphatidylcholine (PC) (Xiao et al., 2010). As the phloem exudate of Arabidopsis has been reported to contain various lipids, such as phospholipids and free FAs (Madey et al., 2002; Guelette et al., 2012), and *AtACBP3pro::GUS* had been detected in the phloem (Zheng et al., 2012), an investigation was initiated to explore the biological function of AtACBP3 in phloem lipid homeostasis and the plant wound responses. Our results suggest that AtACBP3 is a phloem-mobile and wound-inducible protein that plays a role in balancing the composition of polyunsaturated C18-unsaturated FAs and jasmonate in the phloem.

## Materials and Methods

### Plant Materials and Growth Conditions

The Arabidopsis *acbp3* mutant (stock no. SALK_012290, ecotype Col-0) contains a single T-DNA insertion on Southern blot analysis (Chan, 2004), and it expressed non-translatable truncated *AtACBP3* mRNAs as verified by northern blot analysis and western blot analysis (Xiao et al., 2010). Arabidopsis seeds of the *acbp3* mutant, *AtACBP3*-RNAi lines (ecotype Col-0; Xiao et al., 2010), *AtACBP3*-OE and wild-type Col-0 were surface-sterilised in diluted bleach (Clorox) solution [final concentration 1.25% (w/v) sodium hypochlorite] and 1% (v/v) Triton X-100 for 30 min, followed by several washes in sterile water. They were subsequently germinated on Murashige and Skoog (MS) medium. They were stratified for 2 days at 4°C in darkness before germination at 21°C. Plates were incubated in a tissue culture room and seedlings were subsequently potted in soil and grown at 23°C/21°C (day/night) cycles with a day length regime of 16-h light and 8-h dark.

### Collection of Phloem Exudate

Phloem exudates were collected 2–3 h after the onset of light as previously described (Guelette et al., 2012). Fifteen Arabidopsis rosette leaves with petioles from 5- to 6-week-old plants were excised and the cuttings were immediately immersed in ethylenediaminetetraacetic acid (EDTA) solution (20 mM K_2_-EDTA, pH 7.0). Subsequently, the petioles were recut to the same length and quickly immersed into 1.3 mL of the EDTA solution. Leaves were then kept in an opaque and humid container. After 1 h, petioles were rinsed in sterile water to remove residual EDTA and returned to the sterile water. Exudates were collected for 6–8 h under a dark humid condition. Phloem RNA was prepared by adding 100 U of RNase inhibitor (Roche) per 1 mL of deionized water before collection of phloem exudates. The phloem exudates were flash-frozen in liquid nitrogen, lyophilised and stored at -80°C until use.

### Reverse Transcription Polymerase Chain Reactions (RT-PCR) Using Phloem mRNA

Phloem mRNA was extracted using the RNeasy Mini Kit (Qiagen) to detect *AtACBP3* (At4g24230) expression in the RT-PCR analysis. According to Guelette et al. (2012), the mRNA of *UBC9* (At4g27960) was selected as the positive internal control, because it is known to be present in phloem exudates. The negative internal controls were *rbcL* (AtCg00490) and *rbcS* (At5g38410), corresponding to the transcripts of the Rubisco large and small subunits, respectively. The two transcripts (*rbcL* and *rbcS*) were chosen as negative controls in the RT-PCR analysis of phloem exudates, as two independent studies (Deeken et al., 2008; Guelette et al., 2012) have confirmed their absence in phloem exudates. Total leaf mRNA similarly extracted from the 4-week-old wild type was used as a positive external control in RT-PCR. Following DNase treatment (Promega), mRNA (200 ng) was reverse-transcribed using the Omniscript RT Kit (Qiagen). The cDNA from phloem exudates and leaf mRNA samples was used as templates in PCR mixes with *Taq* DNA polymerase (Promega) for amplification under 40 cycles of 94°C, 30 s; 53°C, 30 s, and 72°C, 30 s using primers of *UBC9*, *rbcL*, *rbcS*, and *AtACBP3* (Supplementary Table S1). Equal volumes of PCR products were loaded onto a 2% agarose gel for electrophoresis.

### Micrografting of Arabidopsis

Micrografting experiments were carried out according to Turnbull et al. (2002) with modifications. Seeds of Col-0, *AtACBP3-*OEs and the *acbp3* mutant were surface-sterilised and sown onto MS plates. MS plates were orientated vertically at 4°C for 4 days for seed stratification, and subsequent germination was carried out at 23°C/21°C (day/night) cycles with a regime of 16-h light and 8-h dark. The micrografting experiment was performed using 7-day-old seedlings. Upper hypocotyls of seedlings containing shoot apical meristem and cotyledons were excised from whole seedlings with a surgical blade and re-aligned with lower hypocotyls, which were separated in the same manner. All grafted plants were examined before planting on soil. Grafted plants that had adventitious roots at the grafting junctions were discarded. Grafted plants with no adventitious roots were grown vertically for 7 days in Petri dishes in a humid environment. Successful reunions were transferred onto soil and grown as normal plants. Phloem exudates from 6-week-old scion (rosette leaf) or rootstock tissues were harvested for protein extraction after micrografting.

### Western Blot Analysis

Phloem exudates collected from the 5-week-old wild type and the *acbp3* mutant were directly used for western blot analysis. Sterile water was added into the lyophilised exudates. Protein concentration was determined according to Bradford (1976) and 30 μg of protein were loaded for sodium dodecyl sulphate polyacrylamide gel electrophoresis (SDS-PAGE), followed by protein transfer to polyvinylidene fluoride membranes (GE Healthcare) using the Trans-Blot Cell (Bio-Rad) at 25 V for 20 min. The rabbit polyclonal anti-AtACBP3 antibodies were raised against a synthetic peptide corresponding to amino acids 59 to 72 of AtACBP3 (DARVLESQRNFQVV) (Xiao et al., 2010). The antibodies were preabsorbed overnight in *acbp3* mutant protein extract to remove unspecific binding prior to use (Supplementary Figure S1). The blots were incubated with the preabsorbed antibodies. Detection of immuno-reactive signals was performed using the Amersham ECL Prime Western Blotting Detection Reagent according to the manufacturer’s instruction.

### Immunoelectron Microscopy (IEM)

Five-week-old stems were fixed in a solution of 2% paraformaldehyde and 1.25% glutaraldehyde in 50 mM piperazine-*N,N*-bis (2-ethanesulfonic acid) (pH 7.2) for 1 h under vacuum and then further incubated at 4°C overnight with gentle shaking. The tissues were dehydrated in a graded ethanol series, infiltrated in stepwise increments of London Resin White (London Resin Company) and polymerised at 65°C for over 24 h (His et al., 2001).

Immuno-gold labelling was carried out according to Ye et al. (2016b) with modifications. Ultra-thin sections (80 nm) were prepared and mounted on nickel grids. Grids were blocked on the liquid surface of 30 μL blocking solution droplets containing 1% bovine serum albumin (BSA) and 1% TTBS (20 mM Tris, 500 mM NaCl, 0.1% Tween-20, pH7.5) for 1 h at room temperature and subsequently incubated in anti-AtACBP3 antibodies diluted 1:50 (v/v) at 4°C overnight. In the negative controls, the primary antibodies were replaced with blocking solution or preimmune serum diluted 1:50 (v/v). After three 15-min washes with blocking buffer, the grids were incubated with a 1:100 dilution of anti-rabbit antibodies conjugated to 10 nm gold (Sigma–Aldrich) at room temperature for 1 h. Grids were washed twice in TTBS-BSA, once in TTBS and sterile water, and each rinse lasted 15 min. The grids were subsequently stained with 2% uranyl acetate for 1 min at room temperature and photographed under a Philips 100CM TEM immunoelectron microscope at 80 kV.

Images in **Figure 2** and other images collected from the same experiment were analysed for gold labelling densities in different cell types according to Lee et al. (2011) with modifications. All raw images in this analysis are available in Supplementary IEM Images. Total number of gold particles and total area from each cell type [CC (*n* = 7), SE (*n* = 7) and extracellular space (ES) (*n* = 7)] were counted and measured in Adobe Photoshop CC 2015. The density of gold particles from each cell type is equal to the total number of gold particles divided by its total area (μm^2^).

### Wound Treatment of Plants

Two days prior to any treatment, plants were transferred to 24-h constant light condition to eliminate the induction of AtACBP3 by darkness (Xiao et al., 2010; Zheng et al., 2012). Wound treatment was carried out according to Farmer et al. (2013) and Mousavi et al. (2013). Five-week-old plants were wounded by gently crushing leaf 8 of the plants with a pair of forceps (Farmer et al., 2013). Tissues of locally wounded [leaf 8; Mousavi et al. (2013)] and systemic [leaf 13; Mousavi et al. (2013)] leaves were collected 1 h post wounding (hpw), 2 and 4 hpw for relative gene expression analysis. For quantitative GUS assays, all leaves were wounded and total proteins were extracted. For quantitative real-time polymerase chain reactions (qRT-PCR), unwounded plants (Col-0, *acbp3*, and *AtACBP3-*RNAi) were used as baseline control to monitor the gene expression levels, while gene expression in wounded *acbp3* and *AtACBP3-*RNAi plants were compared with those in wounded Col-0 treated under the same conditions.

### Quantitative and Histochemical GUS Assays

Quantification of GUS activity was carried out by measuring the cleavage of the substrate (Jefferson et al., 1987). The substrate, 4-methylumbelliferyl-β-D-glucuronide (MUG) was purchased from Sigma–Aldrich. Tissues were collected into Eppendorf tubes and frozen in liquid nitrogen, followed by homogenisation. Subsequently, 1 mL GUS Extraction Buffer (150 mM sodium phosphate pH 7.0, 10 mM EDTA, 10 mM β-mercaptoethanol, 0.1% Triton X-100, 0.1% sarcosyl) was added, and samples were gently agitated. The homogenised mixture was centrifuged at 13000 rpm for 15 min. Two hundred μL of supernatant from each sample was used for MUG assays and protein quantification. A 10-μL sample extract and 130-μL Assay Buffer (GUS Extraction Buffer containing 1.2 mM MUG) were mixed and the reaction incubated in the dark at 37°C for 20 min. Subsequently, 10 μL of the reaction was added to 190 μL Stop Buffer (200 mM sodium carbonate) in a 96-well plate. Fluorescence excited at 355 nm was quantified on a plate reader (BMG LABTECH) at 460 nm. A standard curve corresponding to 0, 0.25, 0.5, 2.5, 5, 25, and 50 μM 4-methylumbelliferone (MU) was included for calculation of liberated MU. The fluorescence assay measurements were normalised for protein concentration and recorded as nmoles MU min^-1^ mg^-1^ protein.

For histochemical GUS assay (Jefferson et al., 1987), Arabidopsis leaves was submerged in X-Gluc solution [1 mg mL^-1^ *X*-Gluc, 100 mM sodium phosphate, pH 7.5, 2 mM K_3_Fe(CN)_6_, 2 mM K_4_Fe(CN)_6_, 0.1% Triton X-100] and infiltrated by vacuuming for 1 h, followed by a 4-h incubation at 37°C in darkness. Chlorophyll removal was performed by using 70% ethanol. Samples were then placed above a light-box for photography.

Two sets of controls were used in both GUS assays; a vector line (pBI101) was used as a negative control, and unwounded *AtACBP3pro::GUS* transgenic plants served as a baseline control in GUS expression.

### Quantitative Real-Time Polymerase Chain Reactions (qRT-PCR)

Total leaf mRNA was extracted from 5-week-old Arabidopsis using the RNeasy Mini Kit (Qiagen) followed by DNase (Promega) treatment. First-strand cDNA was synthesised using the Superscript First-Strand Synthesis System (Invitrogen). Subsequently, qRT-PCR were carried out using the FastStart Universal SYBR Green Master Mix (Roche) on a StepOne Plus Real-Time PCR System (Applied Biosystems). Primers of *JASMONATE ZIM-DOMAIN10* (*JAZ10*), *LIPOXYGENASE2* (*LOX2*), *VEGETATIVE STORAGE PROTEIN2* (*VSP2*), *AtACBP3* and *ACTIN2* are shown in Supplementary Table S1. Relative gene expression of *JAZ10* (1 hpw), *LOX2* (2 hpw), *VSP2* (4 hpw), and *AtACBP3* (1 hpw) was normalised to the expression of *ACTIN2.* The duration of wound treatment for analysis of each marker gene (*JAZ10*, *LOX2*, and *VSP2*) was according to Glauser et al. (2009) and Mousavi et al. (2013). Three independent biological repeats were performed to analyse the relative mRNA expression.

### FA Analysis by Gas Chromatography- Mass Spectrometry (GC-MS)

FA analysis was conducted following Carvalho and Malcata (2005) with modifications. Phloem exudates collected and pooled from 150 leaves were dissolved in a transmethylation solution containing 1 mL of toluene, 2 mL of 1% (v/v) sulphuric acid in methanol together with 5 μL of an internal standard [C19:0 (1 mg mL^-1^ hexane)]. The transmethylation solution containing phloem exudates was incubated for 12 h at 50°C for the transmethylation of FAs. After several washes with 5% (w/v) NaCl and hexane, the hexane layer was washed with 4 mL 2% (w/v) KHCO_3_ and then transferred into another test tube followed by vigorous vortexing. Nitrogen gas was passed through each tube to evaporate the hexane. Then 100 μL of hexane was re-added to the test tube to concentrate the FA residues. One μL of the hexane supernatant, containing the FAs, was analysed by Agilent 6890N equipped with a 5973 Mass Selective Detector (MSD) and a 30 m × 0.250 mm DB-WAX column (0.25 μm in film thickness). The samples were positioned and then automatically injected into the column. For sample detection, the oven temperature was increased from 100 to 230°C with a rate of 4°C min^-1^ and postrun at 235°C for 4 min. The GC/MSD On-line Data Analysis was used for data processing after data acquisition; the FAME library was used for compound identification. Quantification of FA species was calculated by comparing the peak area of each FA to the internal standard, followed by normalisation to ng/leaf.

### Isothermal Titration Calorimetry Assays (ITC)

(His)_6_-AtACBP3 expressed from plasmid pAT223 in *Escherichia coli* BL21(DE3) pLysS cells was purified as previously described (Leung et al., 2006). ITC assays were performed using a MicroCal iTC200 system (GE Healthcare). Acyl-CoA esters (C12:0-, C14:0-, C16:1-, C17:0-, C18:2-, and C18:3-CoA esters) were purchased from Avanti Polar Lipids. The 600 μM acyl-CoA ester in the titration syringe was 20-fold higher than the protein concentration (30 μM) in the reaction chamber. Solutions of acyl-CoA esters and (His)_6_-AtACBP3 were degassed under vacuum. Assays were conducted at 25°C, and injections were initiated after equilibration to baseline stability. The acyl-CoA ester (1.5 μL) was injected 20 times into the reaction chamber and each injection lasted 3.6 s at an interval of 120 s between injections. Immediate mixing was ensured by stirring. Raw data was integrated, corrected for non-specific heat, and analysed using the ORIGIN software (OriginLab). Dissociation constants (*K_D_*) were calculated by non-linear regression fitting the isotherm. Each binding assay was performed at least twice independently.

## Results

### AtACBP3, But Not Its mRNA, Was Detected in Wild-Type Col-0 Phloem Exudates

**FIGURE 1:**
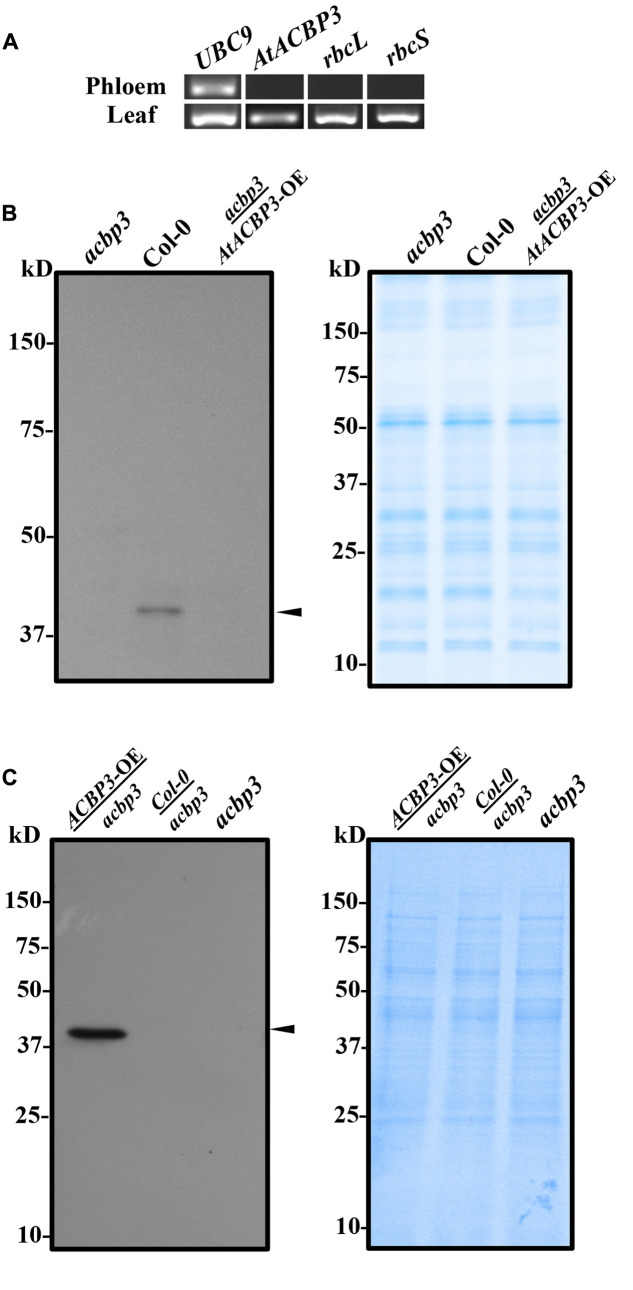
Reverse transcription PCR and western blot analysis of phloem exudates from Arabidopsis. **(A)** Total RNA was collected from phloem exudates and rosette leaves of Col-0 to detect *AtACBP3* and controls (*UBC9*, *rbcL*, and *rbcS*) transcripts. Original images of the analysis are available in Supplementary Figure S2. **(B)** Western blot analysis using anti-AtACBP3 antibodies on 30 μg total phloem exudate proteins from the *acbp3* mutant, wild-type Col-0 and *acbp3* scion grafted on *AtACBP3-*OE rootstock (*acbp3*/*AtACBP3-*OE) (left). A Coomassie Blue-stained gel loaded with the same amount of protein in western blot analysis is shown (right). **(C)** Western blot analysis using anti-AtACBP3 antibodies on 30 μg total root proteins collected from *AtACBP3-*OE*/acbp3*, Col-0/*acbp3, acbp3* (left). A Coomassie Blue-stained gel loaded with the same amount of protein in western blot analysis is shown below (right). Arrowheads indicate the 39-kDa cross-reacting AtACBP3 band. These experiments were repeated twice with consistent results.

Given that *AtACBP3pro::GUS* was localised in the phloem (Zheng et al., 2012), phloem exudates were collected from leaf petioles to verify whether AtACBP3 and/or its mRNA were present in the phloem sap. In RT-PCR analysis, the positive control (*UBC9*) was detected both in the phloem exudates and leaves, indicating that mRNA extraction and RT-PCR analysis were properly performed. Two negative controls (*rbcL* and *rbcS*) were only detectable in leaves but not in phloem exudates (**Figure 1A**), confirming the collection of phloem exudates were free from cellular contaminations of other cell types. Using an *AtACBP3-*specific primer pair (ML1202 and ML1203) (Supplementary Table S1), the 174-bp *AtACBP3* mRNA was not detected in phloem exudate (**Figure 1A**), but western blot analysis showed a strong 39-kDa AtACBP3 cross-reacting band in wild-type phloem exudate (**Figure 1B**). Through micrografting, a 39-kDa AtACBP3 cross-reacting band was also detected in root protein extracts of *acbp3* rootstock micrografted below *AtACBP3-*OE scions while the same cross-reacting band was not detectable in non-grafted *acbp3* roots, indicating that AtACBP3 is mobile from shoot to root (**Figure 1C**). However, no such band was detected in the phloem exudates collected from *acbp3* scions micrografted above the *AtACBP3-*OE rootstocks, implying that AtACBP3 is not mobile from root to shoot (**Figure 1B**).

### AtACBP3 Was Immunolocalised to Companion Cells, Sieve Elements and the Extracellular Space of the Phloem

**FIGURE 2:**
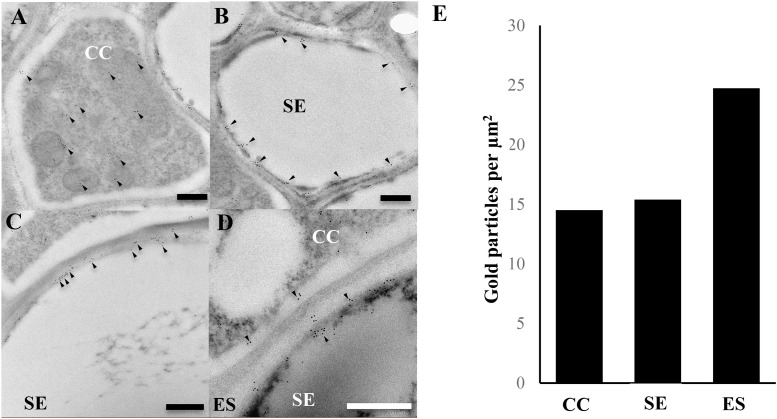
Immunogold localisation of AtACBP3 using anti-AtACBP3 antibodies in transmission electron microscopy of cross sections of apical stems from 5-week-old Arabidopsis. **(A)** Companion cell. **(B)** Sieve element. **(C,D)** Extracellular space. **(E)** Densities of gold particles were quantified per μm^2^. Arrowheads indicate gold particles. Multiple floral stems from different individual plants sectioned and analysed in this experiment showed consistent results. Further images are available in Supplementary IEM Images. CC, companion cell; ES, extracellular space; SE, sieve element. Scale bar = 0.5 μm.

To further investigate the localisation of AtACBP3 in the phloem, IEM was carried out after preabsorption of antibodies (Supplementary Figure S1). Immunogold particles were localised in the phloem (**Figure 2**), particularly in the cytosol of CC (**Figure 2A**) and SE (**Figure 2B**). Immunogold particles were also detected in ES (**Figures 2C,D**), but not in the control incubated with blocking solution (Supplementary Figure S3). A quantitative analysis of gold particle densities showed that the labelling was localised more preferably to ES in comparison to CC and SE (**Figure 2E**).

### *AtACBP3* Expression Was Induced by Mechanical Wounding

**FIGURE 3:**
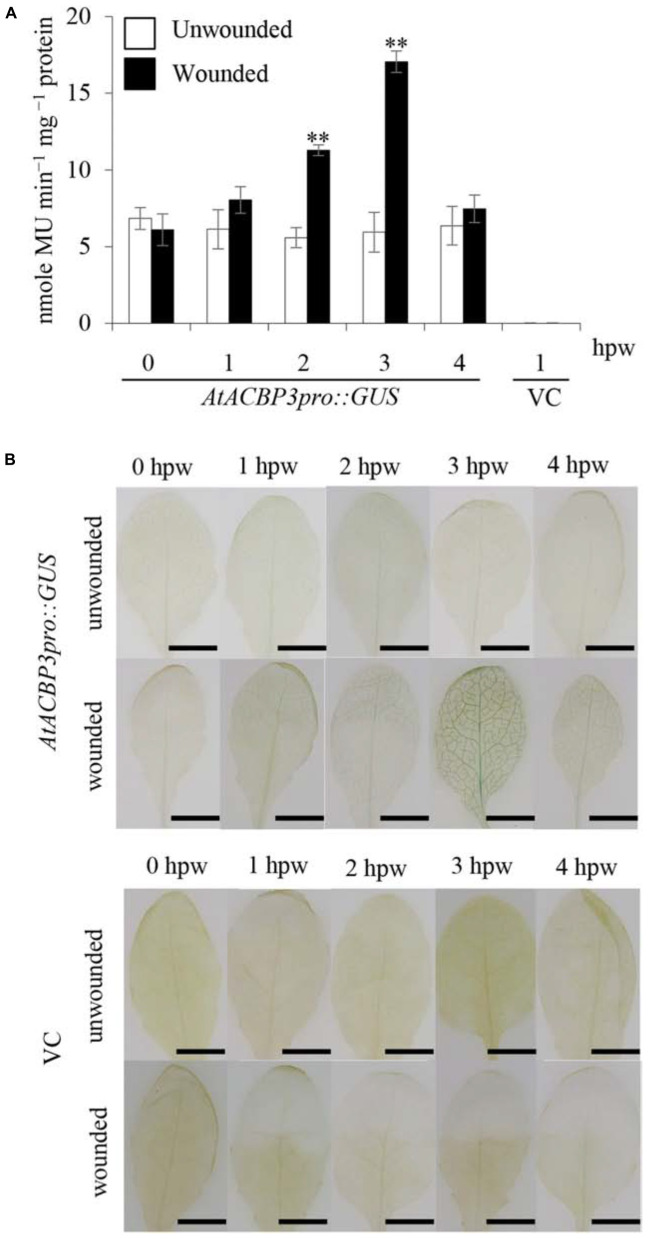
GUS activity assays of transgenic Arabidopsis *AtACBP3pro::GUS* plants following wounding. **(A)** Four-week-old *AtACBP3pro::GUS* transgenic plants were wounded with a pair of forceps and harvested at 1, 2, 3, and 4 h post wounding (hpw) for quantitative GUS activity measurements. Vector (pBI101.3)-transformed plants were analysed at 1 hpw and served as a negative control, while unwounded *AtACBP3pro::GUS* plants were used as a baseline control. “^∗∗^” indicates statistically significant difference (*P* < 0.01, *n* = 3 by Student’s *t-*test) in comparison to unwounded samples collected at the same time point. Error bars represent standard deviations. **(B)** A representation of histochemical staining of wounded 4-week-old leaves from *AtACBP3pro::GUS* and vector (pBI101.3)-transformed (VC) Arabidopsis. These experiments were repeated twice with consistent results. Scale bars = 0.8 cm.

To investigate the effect of wounding on the expression of *AtACBP3*, *AtACBP3pro::GUS* plants were utilised in quantitative and histochemical GUS assays. The results of GUS activity using 4-week-old transgenic plants, 1–4 hpw, showed that *AtACBP3pro::GUS* expression peaked at 3 hpw in comparison to unwounded *AtACBP3pro::GUS* (**Figure 3A**). Histochemical staining of *AtACBP3pro::GUS* also confirmed that AtACBP3 expression was induced by wounding with the highest expression at 3 hpw, while the wounded vector line showed no expression (**Figure 3B**).

### The *acbp3* Mutant Was Less Responsive to Wound in Local and Distal Leaves

**FIGURE 4:**
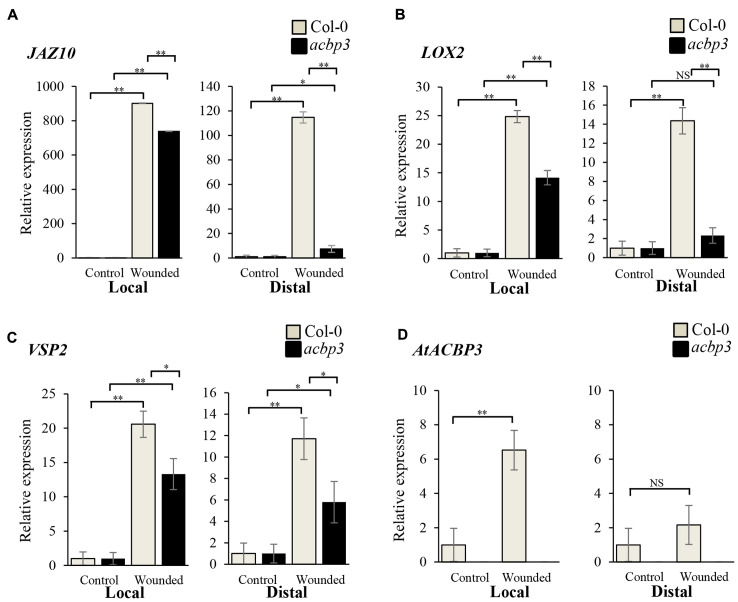
Relative expression of *AtACBP3* and marker genes in the Arabidopsis jasmonate pathway after wounding. Rosette leaves harvested from 5-week-old Col-0 and *acbp3* were leaf 8 (wounded locally) and leaf 13 (distally wounded at leaf 8). Numbering of rosette leaves was according to Farmer et al. (2013). **(A)** Relative expression of *JAZ10* 1 h post wounding (hpw) following Mousavi et al. (2013). **(B)** Relative expression of *LOX2* (2 hpw) following Glauser et al. (2009). **(C)** Relative expression of *VSP2* (4 hpw) following Mousavi et al. (2013). **(D)** Relative expression of *AtACBP3* 1 hpw. Error bars represent standard deviation in each analysis. Square brackets indicate which two groups were compared using the Student’s *t-*test. ^∗∗^*P* < 0.01, *n* = 3; ^∗^*P* < 0.05, *n* = 3; NS, not significant, *n* = 3; Local, wounded leaf 8; Distal, leaf 13 distal to wounded leaf 8. These experiments were repeated twice with consistent results.

The role of *AtACBP3* in the wound response was investigated by comparing wild type, *acbp3* and *AtACBP3-*RNAi leaves in qRT-PCR assays examining *JAZ10, LOX2 and VSP2* expression, as they are deemed to be robust wound-responsive JA marker genes (Bell et al., 1995; Liu et al., 2005; Yan et al., 2007). The expression of *JAZ10*, *LOX2, VSP2* was drastically elevated 1, 2, and 4 hpw in the wild type (**Figures 4A–C**), while their expression was significantly reduced in *acbp3* and *AtACBP3-*RNAi (**Figures 4A–C** and Supplementary Figure S4). Furthermore, the expression of the marker genes was higher in wild-type than either *acbp3* or *AtACBP3-*RNAi distal leaves, indicating that the wound response was adversely affected in *acbp3* and *AtACBP3-*RNAi lines. Another line of evidence on the wound-inducibility of *AtACBP3* was reflected in the upregulation of its mRNA (approximately five times higher at 1 hpw in locally wounded leaves), in comparison to little expression in the distal leaves (**Figure 4D**).

The differences in expression of the three marker genes before and after wounding were also investigated in an *acbp3* complemented line [*acbp3-*C1; generated using a *35S::AtACBP3* construct as verified by Xiao et al. (2010)]. However, *acbp3-*C1 did not show the same extent of induction of the three marker genes in wounded leaves as in the wild type, and the induction of *JAZ10* and *VSP2* was significantly upregulated only in distal leaves (Supplementary Figure S5). When comparing the innate expression level of *JAZ10, LOX2*, and *VSP2* between *acbp3-*C1 and wild type, it was noted that the expression of *JAZ10* in unwounded *acbp3-*C1 was 15 times that of the wild type. However, the expression of *LOX2* and *VSP2* decreased when compared to the wild type (Supplementary Figure S6).

### Reduced Levels of Defence-Related FAs Accumulate in *acbp3* Mutant and *AtACBP3*-RNAi Lines

**FIGURE 5:**
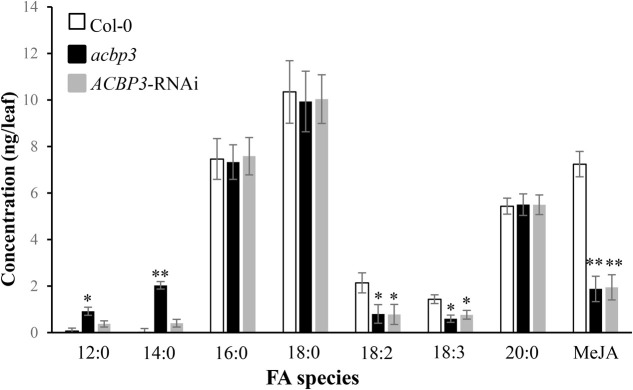
Quantitative gas chromatography-mass spectrometry analysis of fatty acids from phloem exudates of 5-week-old wild-type Col-0 Arabidopsis, *acbp3* and *AtACBP3-*RNAi lines. Error bars represent standard deviation (*n* = 3) in each analysis. The Student’s *t-*test was used for statistical analyses. “^∗^” indicates a statistically significant (*P* < 0.05) elevation or reduction in comparison with the wild-type Col-0. “^∗∗^” indicates a very statistically significant different (*P* < 0.01) elevation or reduction in comparison with the wild-type Col-0. These experiments were repeated twice with consistent results.

GC-MS analysis of phloem exudates from the wild-type Col-0, the *acbp3* mutant as well as the *AtACBP3*-RNAi lines revealed that C16:0- and C18:0-FAs were the major FAs in all genotypes (**Figure 5**). Levels of defence-related C18:2-FA and C18:3-FA decreased in *acbp3* and *AtACBP3-*RNAi in comparison to the wild type (**Figure 5**). Also, a decrease in methyl jasmonate (MeJA) in both *acbp3* and *AtACBP3-*RNAi and an accumulation of saturated C12- and C14-FAs in *acbp3* were observed in comparison to the wild type (**Figure 5**).

### Recombinant (His)_6_-AtACBP3 Binds Defence-Related Acyl-CoA Esters *In Vitro*

**FIGURE 6:**
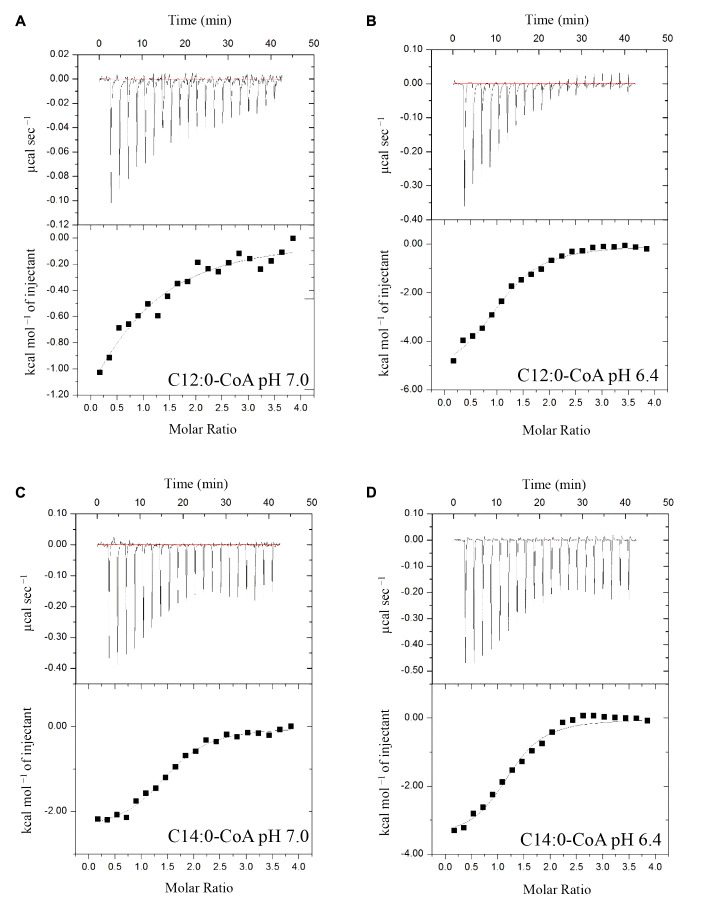
Binding isotherms of recombinant (His)_6_-AtACBP3 titrated with C12:0- and C14:0-CoA esters at 25°C in isothermal titration calorimetry. The panel shows raw data of 30 μM recombinant (His)_6_-AtACBP3 titrated with 600 μM of C12:0-CoA ester, pH 7.0 **(A)**; C12:0-CoA ester, pH 6.4 **(B)**; C14:0-CoA ester, pH 7.0 **(C)** and C14:0-CoA ester, pH 6.4 **(D)**. Each assay had three technical repeats and was repeated at least twice, each using independently prepared (His)_6_-AtACBP3.

**FIGURE 7:**
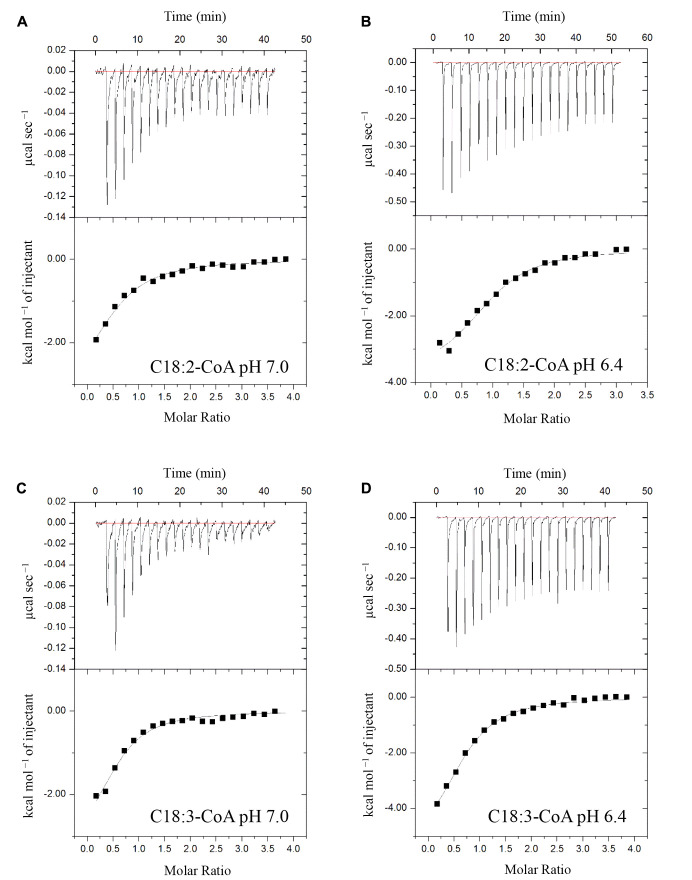
Binding isotherms of recombinant (His)_6_-AtACBP3 titrated with C18:2- and C18:3-CoA esters at 25°C in isothermal titration calorimetry. The panel shows raw data of 30 μM recombinant (His)_6_-AtACBP3 titrated with 600 μM of C18:2-CoA ester, pH 7.0 **(A)**; C18:2-CoA ester, pH 6.4 **(B)**; C18:3-CoA ester, pH 7.0 **(C)** and C18:3-CoA ester, pH 6.4 **(D)**. Each assay had three technical repeats and was repeated at least twice, each using independently prepared (His)_6_-AtACBP3.

ITC assays were next performed to expand on the *in vitro* binding profile of AtACBP3 with acyl-CoA esters. A range of medium- and long-chain acyl-CoA esters corresponding to the FAs identified in the phloem exudates were selected for analysis. The binding isotherms of recombinant (His)_6_-AtACBP3 titrated with C12:0-, C14:0-, C18:2-, and C18:3-CoA esters at 25°C are displayed in **Figures 6**, **7**. ITC assays were carried out at two different pH values, pH 7.0 (**Figures 6A,C**, **7A,C**) and pH 6.4 (**Figures 6B,D**, **7B,D**), because AtACBP3 is expected to be subjected to an acidic pH when targeted to the apoplast and the phloem (Gao et al., 2004; Leung et al., 2006; Hijaz and Killiny, 2014). All four acyl-CoA esters were shown to bind (His)_6_-AtACBP3 *in vitro*, with *K_D_* values in the micromolar range (**Figures 6**, **7**). The assays using C12:0- and C18:3-CoA esters also indicated lower *K_D_* values at pH 6.4 in comparison to pH 7.0 (**Table 1**). However, (His)_6_-AtACBP3 did not bind MeJA (Supplementary Figure S8), suggesting that AtACBP3 is not likely to transport MeJA directly in the phloem.

**Table 1:** Dissociation constants between (His)_6_-AtACBP3 and acyl-CoA esters.

Acyl-CoA esters	pH	*K_D_* (μM)	*n* (kcal/mol)
C12:0	7.0	33.1 ± 13.0	1.0 ± 0.3
C12:0	6.4	6.1 ± 0.9	1.0 ± 0.0
C14:0	7.0	4.0 ± 0.6	1.5 ± 0.0
C14:0	6.4	3.3 ± 0.8	1.2 ± 0.0
C16:1	7.0	5.1 ± 1.9	1.2 ± 0.1
C16:1	6.4	9.7 ± 2.1	1.63 ± 0.0
C17:0	7.0	21 ± 4.7	1.8 ± 0.1
C17:0	6.4	3.7 ± 1.1	2 ± 0.0
C18:2	7.0	13.4 ± 1.4	0.5 ± 0.0
C18:2	6.4	6.4 ± 1.0	0.9 ± 0.0
C18:3	7.0	8.6 ± 2	0.6 ± 0.0
C18:3	6.4	9.0 ± 0.1	0.7 ± 0.0


*Each assay had three technical repeats and was repeated at least twice using (His)_*6*_-AtACBP3 that was independently prepared. Standard deviations were calculated based on readings of three technical repeats.*

## Discussion

### AtACBP3 Is Likely an Apoplastic Phloem-Mobile Protein

As mature SEs are enucleated, it is likely that the AtACBP3 protein which was detected in phloem exudates on western blot analysis (**Figure 1**), is synthesised in the neighbouring CCs or immature SEs and subsequently exported to mature SEs. Indeed, IEM localised AtACBP3 to both cell types (**Figure 2**). Detection of AtACBP3 in the apoplast is consistent with previous observation that AtACBP3 is targeted extracellularly (Leung et al., 2006). Together with the detection of AtACBP3 in wild-type phloem exudate (**Figure 1B**) and the promoter activity of *AtACBP3* in the root vasculature of transgenic Arabidopsis seedlings (Supplementary Figure S9) and 32-day-old mature plants (Zheng et al., 2012), the detection of AtACBP3 in the roots of grafted plants is not surprising and further suggests that AtACBP3 can be transported from shoot to root (**Figure 1C**).

Proteins previously identified in the phloem include signalling molecules in flowering time regulation and plant defence (Corbesier et al., 2007; Mathieu et al., 2007; Champigny et al., 2013; Carella et al., 2016) as well as a sucrose/H^+^ symporter, AtSUC2 (Srivastava et al., 2008). Phloem LTPs have been postulated to participate in systemic transport related to SAR in the phloem (Champigny et al., 2013; Barbaglia et al., 2016). AtDIR1 is an apoplastic phloem LTP that was reported to function in long-distance transport related to SAR in the phloem (Maldonado et al., 2002; Champigny et al., 2013). Its ability to interact with lipids was confirmed by observation of its binding to two lysophosphatidylcholines with *K_D_* values in the nanomolar range (Lascombe et al., 2008).

The movement of proteins via plasmodesmata into SEs has been demonstrated by Balachandran et al. (1997) and Ishiwatari et al. (1998). More recently, PLASMODESMATA-LOCALISED PROTEIN5 (PDLP5) was reported to mediate cell-to-cell communication (Lee et al., 2011), and the overexpression of PDLP5 affected the long-distance movement of AtDIR1 (Carella et al., 2015). Although AtDIR1 localises to the apoplast, it may access the phloem via the plasmodesmata, either after cleavage of its signal peptide or from a cytosolic or ER pool, such that AtDIR1 protein accesses the phloem via plasmodesmata during SAR (Champigny et al., 2011). Given the demonstrated association of AtACBP3 with the ER/Golgi complex as well as its apoplastic localisation (Xiao et al., 2010) and involvement in SAR (Xiao and Chye, 2011b; Xia et al., 2012), it is possible that some AtACBP3 protein may be transported symplastically via the plasmodesmata similar to AtDIR1. In fact, another AtACBP, AtACBP6 has been shown to interact with plasmodesmata-localised protein, PDLP8 (Ye et al., 2017) and is localised to the plasmodesmata in the phloem (Ye et al., 2016a). Taken together, these data suggest that an intracellular pool of AtACBP3 may access SEs via the plasmodesmata for long-distance transport in the phloem.

### AtACBP3 May Participate in Jasmonate Production after Wounding

In this study, *AtACBP3* was demonstrated to be locally wound-inducible at 2 hpw and peaked at 3 hpw as shown in **Figure 3**. Depletion of *AtACBP3*, however, impaired the induction of wound-responsive JA marker genes in both local and distal leaves after wounding (**Figure 4**). However, a similar level of induction in marker gene expression was not detected in the complementary line (*acbp3-*C1) (Supplementary Figures S4, S5). This observation in *acbp3-*C1 may be attributed to the use of the *35S* promoter driving *AtACBP3* resulting in an increased amount of endogenous salicylic acid (SA) (Xiao and Chye, 2011b) which may have interfered with JA biosynthesis (discussed in Caarls et al., 2015). Furthermore, C18:3-FA and MeJA accumulation was lower in the phloem exudate of *acbp3* and *AtACBP3-*RNAi excised leaves in comparison to the wild type (**Figure 5**). The rapid accumulation of jasmonate in distal leaves after wounding has been well characterised (Glauser et al., 2008, 2009). But the mechanism(s) on how these wound signals are mobilised systemically remains to be resolved. In tomato and Arabidopsis, it has been suggested that jasmonate itself is a systemic signal (Li et al., 2002; Thorpe et al., 2007; Sato et al., 2011; Chauvin et al., 2013; Gasperini et al., 2015). On the other hand, a competing theory suggests that JA-related leaf-to-leaf wound signalling and the systemic biosynthesis of JA are facilitated by electric signals known as wound-activated surface potential changes (Mousavi et al., 2013).

To this end, there are two possibilities on the role of AtACBP3 in jasmonate biosynthesis in the phloem. In Arabidopsis, PC homeostasis is crucial for plastidic galactolipid biosynthesis (Warwick et al., 1986; Wang et al., 2014). Upon wounding, free C18:3-FA is released from plastid membranes (Narváez-Vásquez et al., 1999; Ishiguro et al., 2001) and its content increases in leaves (Conconi et al., 1996), thereby providing the precursors for JA biosynthesis (for a review see Koo and Howe, 2009). *AtACBP3-*OEs have been reported to have markedly lower PC but higher galactolipid, including arabidopside, content than the wild type (Xiao et al., 2010), suggesting that AtACBP3 may participate in the transition of extraplastidic lipids from PC to galactolipids. Depletion of AtACBP3 in the phloem in Arabidopsis rosettes supports its involvement in the homeostasis of extraplastidic lipid exchange (Xiao et al., 2010) in the phloem and production of JA upon or after wounding (**Figure 5**). Another possibility is that AtACBP3 regulates arabidopsides in the phloem as they have been widely postulated to function in wounding and JA production (Stelmach et al., 2001; Hisamatsu et al., 2003, 2005; Andersson et al., 2006; Buseman et al., 2006; Kourtchenko et al., 2007). Wounding and insect infestations can trigger the accumulation of arabidopsides and other 9-LOX–derived oxylipins in Arabidopsis (Buseman et al., 2006; Nalam et al., 2012), and *AtACBP3-*OEs have been shown to overaccumulate arabidopsides (Xiao et al., 2010).

A recent study also demonstrated that blockage of plastidic galactolipid biosynthesis causes overproduction of arabidopsides and leads to abnormal phloem cap lignification in Arabidopsis (Lin et al., 2016). Although little is known about the exact functions of these oxygenated galactolipids in relation to the biosynthesis of free oxylipins at a cellular level, the accumulation of MeJA in phloem exudates collected from excised leaf petioles (**Figure 5**) and arabidopsides in *AtACBP3*-OEs (Xiao et al., 2010) strengthened the role of arabidopsides in wounding and jasmonate biosynthesis. Although it would be interesting to investigate the jasmonate content in phloem exudates collected from excised leaf petioles after wounding the leaves, transcripts of *rbcS* and *rbcL* can be detected in the phloem exudates collected after wounding (data not shown), indicating the phloem exudates collected after wounding contained cellular contamination from the wounded tissues, thereby preventing the analysis of jasmonate content in phloem exudates after wounding.

C12:0- and C14:0-FA accumulation in *acbp3* phloem exudates, but not in the *AtACBP3-*RNAi lines or the wild type (**Figure 5**), may have arisen from complete loss of AtACBP3 function in *acbp3* but a partial loss in the *AtACBP3*-RNAi lines [as verified in northern blot analysis by Xiao et al. (2010)]. In canola plants, sublethal UV irradiation caused an 11-fold increase in C12:0-FA and moderate accumulation of C14:0-FA in the phloem (Madey et al., 2002). Herein, the accumulation of C12:0- and C14:0-FAs in *acbp3* phloem exudates bears resemblance to similar accumulation following response to UV irradiation in canola phloem sap (Madey et al., 2002).

### AtACBP3 Potentially Binds Fatty-Acyl-CoA Esters in the Phloem

Previous *in vitro* Lipidex binding assays had shown that (His)_6_-AtACBP3 binds to C16:0-, C18:1- and C20:4-CoA esters (Leung et al., 2006). In this study, ITC demonstrated that (His)_6_-AtACBP3 not only binds medium-chain (C12:0- and C14:0-) but also long-chain (C16:1-, C17:0-, C18:2-, and C18:3-) acyl-CoA esters (**Figures 6**, **7** and Supplementary Figure S7). Some of their FA derivatives (C12:0-, C14:0-, C16:1-, and C18:2-FAs) have already been identified in Arabidopsis phloem exudate (Guelette et al., 2012). A recent study using microscale thermophoresis (MST) has shown that recombinant AtACBP3 binds long-chain (C18:2- and C20:0-) and very-long-chain (C22:0- and C24:0-) acyl-CoA esters (Xie et al., 2015). Interestingly, the *K_D_* values from our study and Xie et al. (2015) were both within the micromolar range.

An attempt to determine the binding affinity between (His)_6_-AtACBP3 with MeJA in ITC showed that AtACBP3 is unlikely to be a MeJA transporter in the phloem (Supplementary Figure S8). Only recently, did evidence emerge that the Arabidopsis JASMONATE TRANSPORTER1 (AtJAT1), also known as Arabidopsis ABC TRANSPORTER G FAMILY MEMBER16, is a cellular jasmonoyl-isoleucine transporter that mediates nuclear entry of jasmonoyl-isoleucine (Li et al., 2017). Although jasmonate has been considered as a long-distance signal (Li et al., 2002; Thorpe et al., 2007; Sato et al., 2011), the long-distance transporter(s) of jasmonate remains elusive.

**FIGURE 8:**
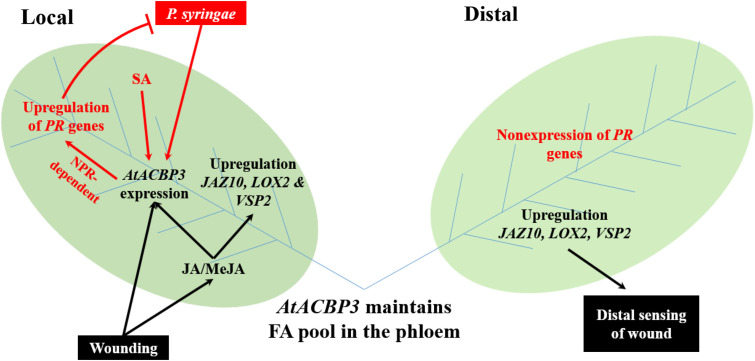
A proposed model of AtACBP3 in plant stress responses was illustrated. Two leaves (labelled Local and Distal) are connected by vascular tissues which include phloem (blue lines) and xylem (not shown). Black colour scheme represents a JA-related pathway. Red colour scheme represents a SA-related pathway. ACC, 1-aminocyclopropane-1-carboxylic acid; *AtACBP3, Arabidopsis thaliana ACYL-COA-BINDING PROTEIN3*; JA, jasmonic acid; *JAZ10, JASMONATE-ZIM-DOMAIN PROTEIN10; LOX2, LIPOXYGENASE2*; MeJA, methyl jasmonate; NPR1, NONEXPRESSOR OF PR GENES1; *PR* genes, *PATHOGENESIS-RELATED* genes; SA, salicylic acid; *VSP2, VEGETATIVE STORAGE PROTEIN2*.

Some proteins that are involved in JA-mediated plant defence are also known to be involved in SA-mediated defence pathways. Noteworthy examples include various fatty-acid desaturases (FAD) which convert C18:2-FA to C18:3-FA, and C18:3-FA is the precursor of jasmonic acid (Zimmerman and Feng, 1978). Arabidopsis *fad3 fad7 fad8* is deficient in C18:3-FA and is hypersensitive to insect infection (McConn et al., 1997). However, a loss-of-function mutation in tomato *fad7* enhanced aphid resistance in a SA-dependent manner (Avila et al., 2012). Interestingly, AtACBP3 appears to be involved in this complex network of JA- and SA-mediated plant defence (**Figure 8**). AtACBP3 is induced by both JA and SA (Xiao and Chye, 2011b). Locally, the overexpression of *AtACBP3* confers upregulation of *PATHOGEN-RELATED* genes and *NONEXPRESSOR OF PR GENES1*-dependent resistance to *P. syringae* (Xiao and Chye, 2011b). In this study, mechanical wounding upregulated the expression of *AtACBP3* and JA marker genes in local leaves. Distally, AtACBP3 is involved in the transport of wound signalling, possibly by complexing with C18:2- and C18:3-acyl-CoA esters, culminating in the upregulation of JA marker genes. However, *PR* genes were not induced in distal tissues when Arabidopsis rosette leaves were infected distally by *P. syringae* (Xiao and Chye, 2011b). Nevertheless, our observations support AtACBP3 participation in the maintenance of a pool of FA/acyl-CoA esters in the phloem, similar to the role of AtACBP6 in the cytosol for jasmonate production (Ye et al., 2016a). Thus, the existence of two AtACBPs in the phloem now provides new evidence on the importance of these proteins in phloem lipid metabolism and plant defence.

## Author Contributions

T-HH and M-LC conceived and designed the experiments. T-HH and S-CL performed and analysed the isothermal titration calorimetry assays. T-HH performed and analysed the fatty acid analysis. M-LC, S-CL, and Z-WY provided critical advice and suggestions on the design of experiments. T-HH performed all other experiments and analysed the data. T-HH, S-CL, Z-WY and M-LC wrote the manuscript.

## Conflict of Interest Statement

The authors declare that the research was conducted in the absence of any commercial or financial relationships that could be construed as a potential conflict of interest.
