# Corrigendum: Depletion of Arabidopsis ACYL-COA-BINDING PROTEIN3 Affects Fatty Acid Composition in the Phloem

**DOI:** 10.3389/fpls.2021.632503

**Published:** 2021-02-16

**Authors:** Tai-Hua Hu, Shiu-Cheung Lung, Zi-Wei Ye, Mee-Len Chye

**Affiliations:** School of Biological Sciences, The University of Hong Kong, Pokfulam, Hong Kong

**Keywords:** acyl-CoA esters, fatty acids, linolenic acid, jasmonate, oxylipins, wounding

In the original article, there was a mistake in Figure 3 as published. The representative images of the unwounded VC at 0 hpw and the unwounded VC at 3 hpw were duplicated. Figure 3B has now been updated by replacing the two duplicated images with new representative images previously taken from the same VC batch in the experiment. The corrected Figure 3 appears below.

**Figure 3:**
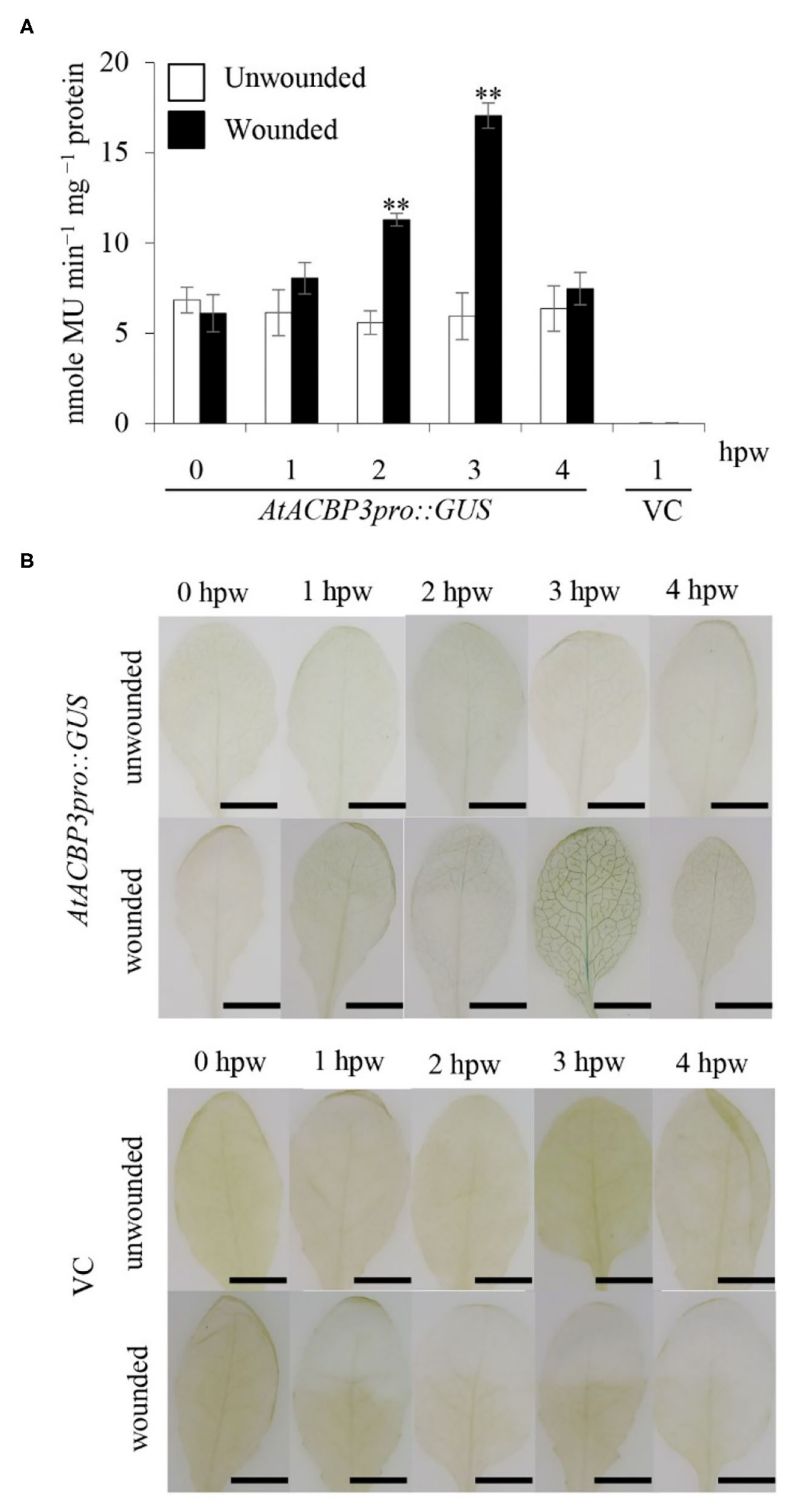
GUS activity assays of transgenic Arabidopsis *AtACBP3pro::GUS* plants following wounding. **(A)** Four-week-old *AtACBP3pro::GUS* transgenic plants were wounded with a pair of forceps and harvested at 1, 2, 3, and 4 h post wounding (hpw) for quantitative GUS activity measurements. Vector (pBI101.3)-transformed plants were analysed at 1 hpw and served as a negative control, while unwounded *AtACBP3pro::GUS* plants were used as a baseline control. “^**^” indicates statistically significant difference (*P* < 0.01, *n* = 3 by Student's *t-*test) in comparison to unwounded samples collected at the same time point. Error bars represent standard deviations. **(B)** A representation of histochemical staining of wounded 4-week-old leaves from *AtACBP3pro::GUS* and vector (pBI101.3)-transformed (VC) Arabidopsis. These experiments were repeated twice with consistent results. Scale bars = 0.8 cm.

The authors apologize for this error and please note that the replaced images do not change the scientific conclusions of the article in any way. The original article has been updated.

