# Supplementary material for: Depletion of Arabidopsis ACYL-COA-BINDING PROTEIN3 Affects Fatty Acid Composition in the Phloem
Source: Front Plant Sci. 2018 Jan 25;9:2. doi: 10.3389/fpls.2018.00002 (PMC5789640; doi:10.3389/fpls.2018.00002)
Supplement: Supplementary file 1 [file Supplementary_Figures_and_Table.PDF]

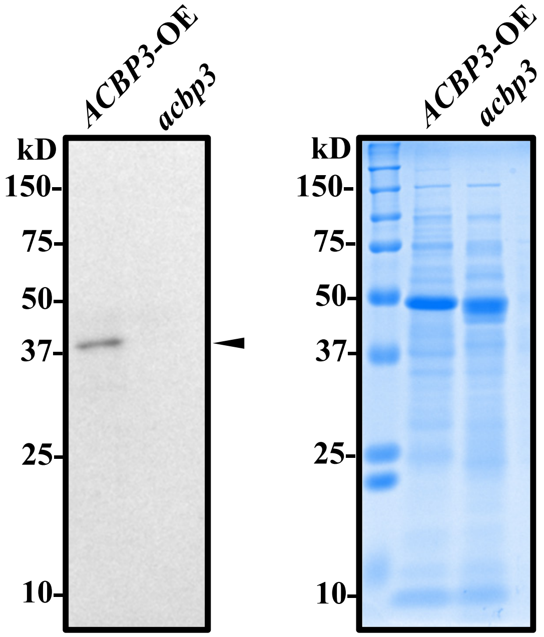

Figure S1 | Western blot analysis showing specificity of the antibodies.

Western blot analysis using anti-AtACBP3 antibodies on 30 μg total leaf proteins from *ACBP3*-OE and *acbp3* shows the antibodies specifically cross-reacted with AtACBP3. A Coomassie Blue-stained gel loaded with the same amount of protein in western blot analysis is also shown. Arrowheads indicate the 39-kDa cross-reacting AtACBP3 band.

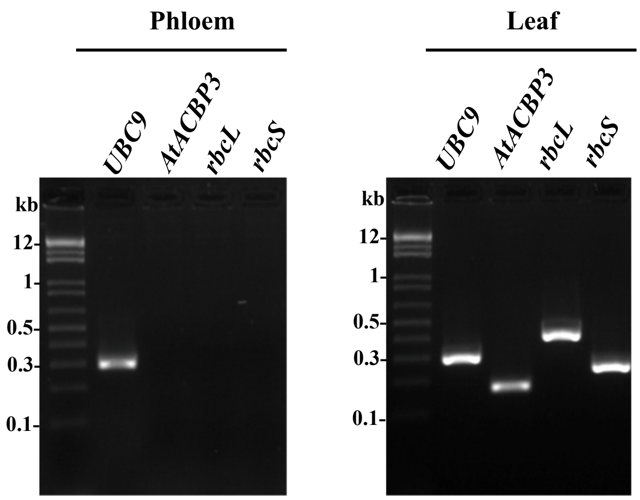

Figure S2 | Original uncropped images of Figure 1A. Total RNA was collected from phloem exudates and rosette leaves of Col-0 to detect *AtACBP3* (174 bp) and controls [*UBC9* (290 bp), *rbcL* (372 bp) and *rbcS* (230 bp)] transcripts. The primers used in this figure are available in Supplementary Table S1. These experiments were repeated twice with consistent results.

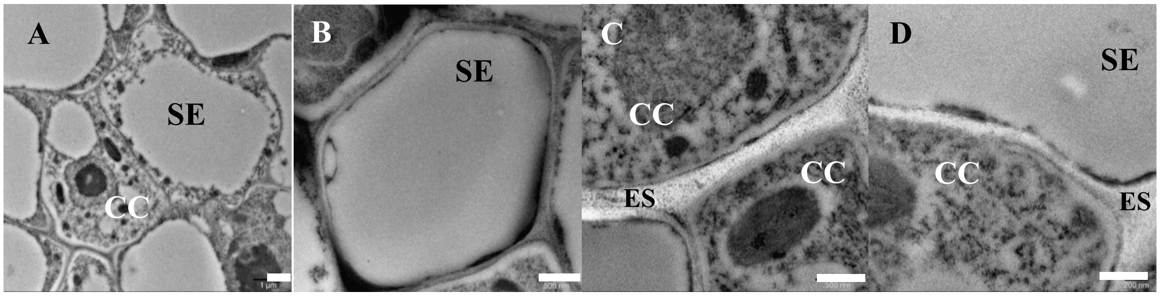

Figure S3 | Immunogold localization of AtACBP3 using blocking solution as a control in transmission electron microscopy of cross sections of apical stems from 5-week-old Arabidopsis.

(A) Whole view of the cross section in the phloem. (B) Sieve element. (C)-(D) Extracellular space of companion cells and sieve elements. CC, companion cell; SE, sieve element; ES, extracellular space. Scale bar = 0.5 μm.

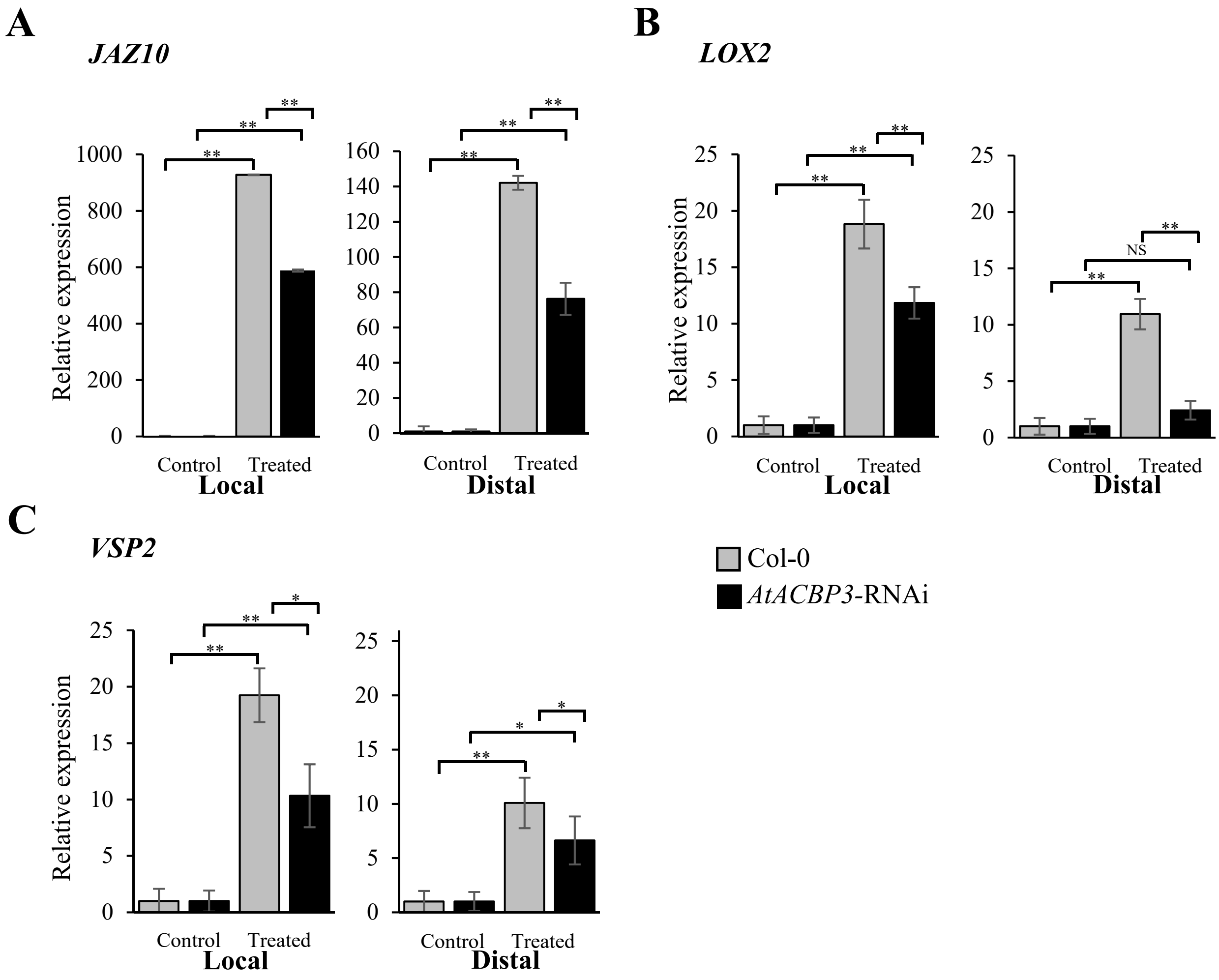

Figure S4 | Relative expression of marker genes in the Arabidopsis jasmonate pathway after wounding. Rosette leaves harvested from 5-week-old Col-0 and *AtACBP3*-RNAi were the local leaf 8 (wounded) and leaf 13 (distally wounded at leaf 8). Numbering of rosette leaves was according to Farmer *et al.* (2013). (A) Relative gene expression of *JAZ10* 1 hour post wounding (hpw) following Mousavi *et al.* (2013). (B) Relative gene expression of *LOX2* (2 hpw) following Glauser *et al.* (2009). (C) Relative gene expression of *VSP2* (4 hpw) following Mousavi *et al.* (2013). Square brackets indicate which two groups were compared using the Student's *t* test. **, $P < 0.01$, n = 3; *, $P < 0.05$, n = 3; NS, not significant, n = 3; Local, wounded leaf 8; Distal, leaf 13 distal to wounded leaf 8. These experiments were repeated twice with consistent results.

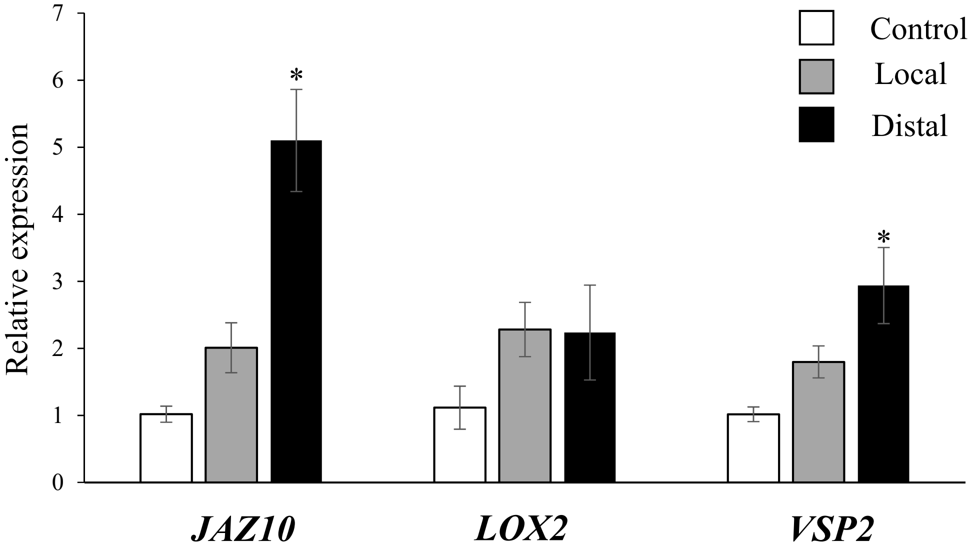

Figure S5 | Relative expression of marker genes in the jasmonate pathway in Arabidopsis. Rosette leaves harvested from the 5-week-old *acbp3*-complemented line were the local leaf 8 (wounded) and leaf 13 (distally wounded at leaf 8). Numbering of rosette leaves was according to Farmer *et al.* (2013). Relative expression of *JAZ10, LOX2* and *VSP2* were analysed according to Glauser *et al.* (2009) and Mousavi *et al.* (2013). The Student's *t* test was used for statistical analyses. *, significantly higher than unwounded control leaves ($P < 0.05$, n = 3). This experiment was repeated twice with consistent results.

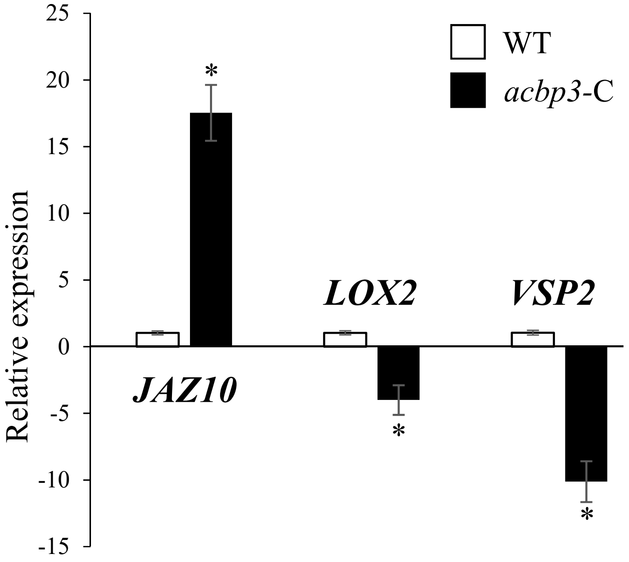

Figure S6 | Relative expression of marker genes in the jasmonate pathway between unwounded *acbp3*-complemented lines (*acbp3*-C) and the wild type (WT). Rosette leaves were harvested from 5-week-old Col-0 and *acbp3*-C for qRT−PCR analysis. The Student's *t* test was used for statistical analyses. H, significantly different from wild type ($P < 0.05$, n = 3). This experiment was repeated twice with consistent results.

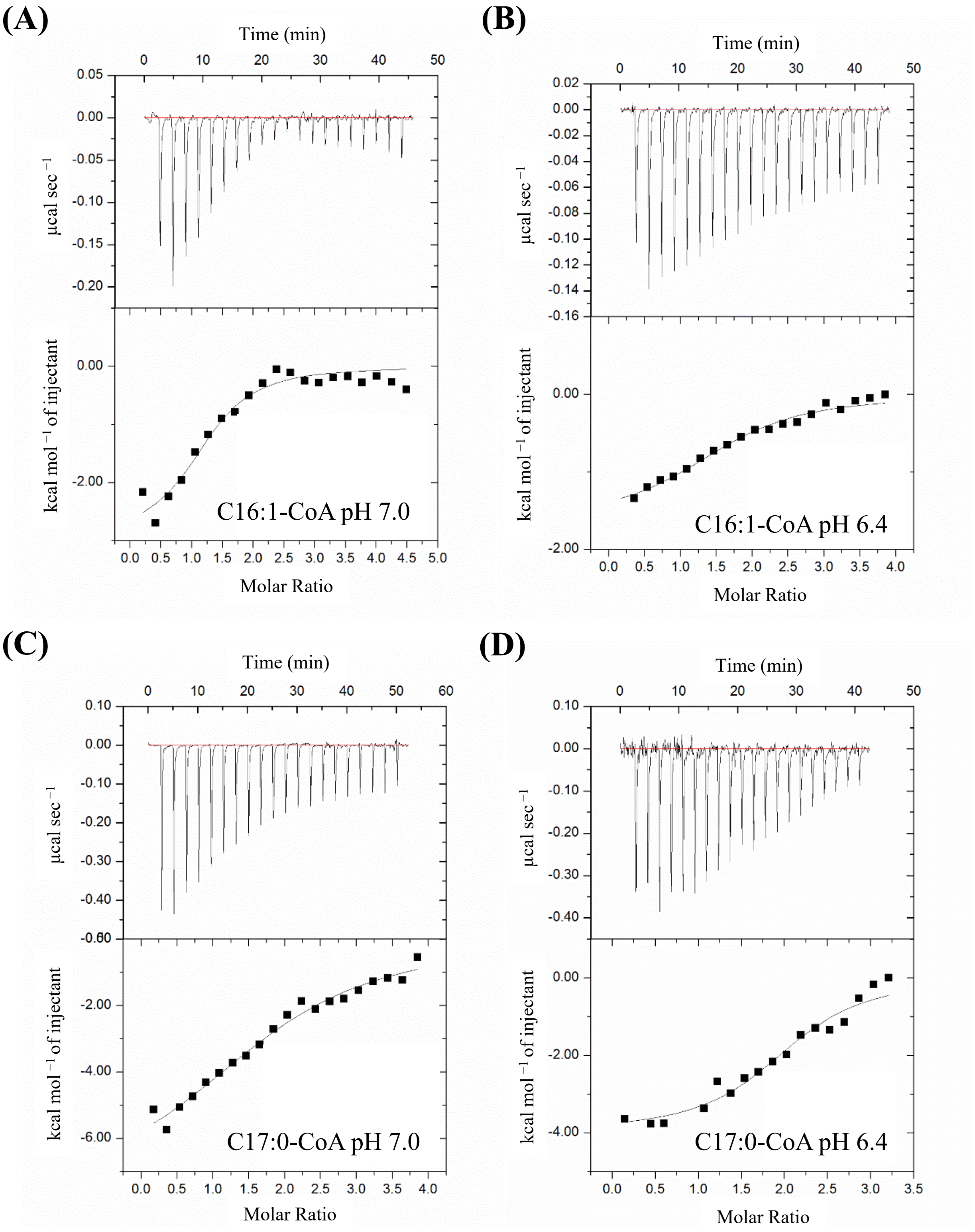

Figure S7 | Binding isotherms of recombinant $(His)_6$-AtACBP3 titrated with C16:1 and C17:0-CoA esters at 25°C in isothermal titration calorimetry.

The panel shows raw data of 30 μM recombinant $(His)_6$-AtACBP3 titrated with 600 μM of C16:1-CoA ester, pH 7.0 (A); C16:1-CoA ester, pH 6.4 (B); C17:0-CoA esters, pH 7.0 (C) and C17:0-CoA ester, pH 6.4 (D). Each assay had three technical repeats and was repeated at least twice, each using independently-prepared (His)6-AtACBP3.

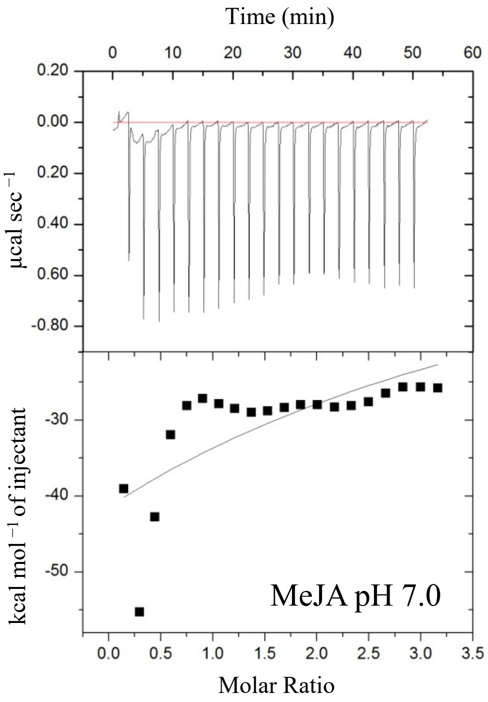

Figure S8 | Binding isotherms of recombinant (His)$_6$-AtACBP3 titrated with saturated methyl jasmonate at 25°C in isothermal titration calorimetry.

The panel shows raw data of 57 μM recombinant (His)$_6$-AtACBP3 titrated with saturated methyl jasmonate (MeJA) at pH 7.0. Each assay had three technical repeats and was repeated at least twice, each using independently-prepared (His)6-AtACBP3.

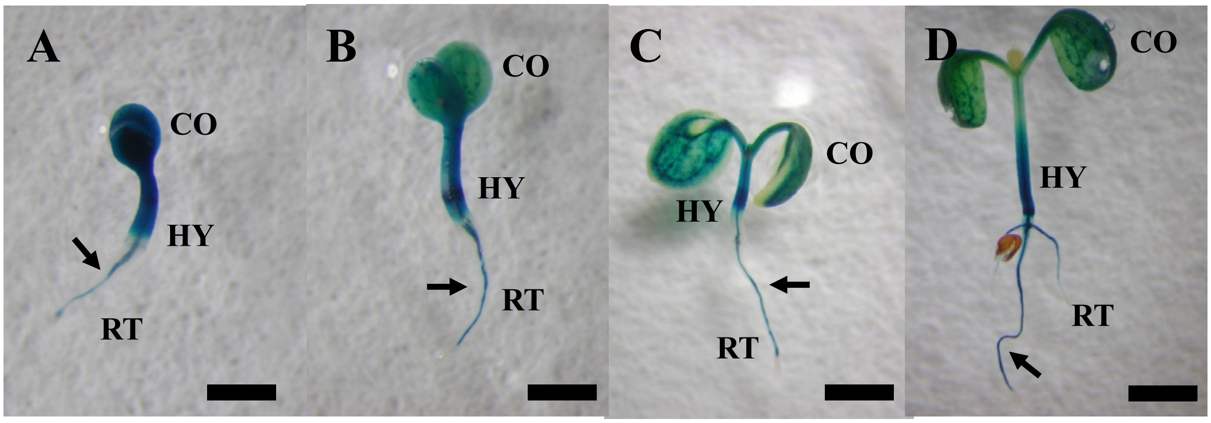

Figure S9 | Spatial and temporal expression of *ACBP3pro::GUS* in transgenic Arabidopsis seedlings. Histochemical GUS staining shows expression of GUS from the *ACBP3* 5’-flanking region in 3-day-old seedling (A); 5-day-old seedling (B); 7-day-old seedling (C) and a 9-day-old seedling (D). CO, cotyledon; HY, hypocotyl; RT, root. Arrows indicate *AtACBP3pro::GUS* expression in roots. Scale bar = 1 mm. The experiment was repeated twice, with three biological repeats analysed in each repeat, all showing consistent results.

Table S1 | Primers used in this study

Name of primers	Sequence	Source
AtACBP3 forward	GACAGGACGAACAGAGCG	Xiao *et al.* (2010)
AtACBP3 reverse	CAGATTCAGCGACTAAGACAT	
JAZ10 forward	ATCCCGATTTCTCCGGTCCA	Mousavi *et al.* (2013)
JAZ10 reverse	ACTTTCTCCTTGCGATGGGAAGA	
LOX2 forward	GCC ATT GAG TTG ACT TGT CC	Grebner *et al.* (2013)
LOX2 reverse	CAC TTA GTT GTC TAT TTG CCG C	
rbcL forward	GCTACCACATCGAGCCCG	Guelette *et al.* (2012)
rbcL reverse	CGGCACAAAATAAGAAACGG	
rbcS forward	CATCCCCGGCTCAGGCC	Guelette *et al.* (2012)
rbcS reverse	AAGGAATCCACTTGTTGCGG	
UBC9 forward	TGGCTTCGAAAAGGATCTTG	Guelette *et al.* (2012)
UBC9 reverse	TCGATATGGTGAGTGCAGGA	
VSP2 forward	CCGTGTGCAAAGAGGCTTA	Mousavi *et al.* (2013)
VSP2 reverse	CACAACTTCCAACGGTCAC	
ACTIN2 forward	CCCGCTATGTATGTCGC	Du *et al.* (2013)
ACTIN2 reverse	AAGGTCAAGACGGAGGAT	

Supplemental references

Du Z.Y., Chen M.X., Chen Q.F., Xiao S. & Chye M.L. (2013) Arabidopsis acyl-CoA-binding protein ACBP1 participates in the regulation of seed germination and seedling development. *Plant Journal*, 74, 294-309.

Grebner W., Stingl N.E., Oenel A., Mueller M.J. & Berger S. (2013) Lipoxygenase6-dependent oxylipin synthesis in roots is required for abiotic and biotic stress resistance of Arabidopsis. *Plant Physiology*, 161, 2159-2170.

Guelette B.S., Benning U.F. & Hoffmann-Benning S. (2012) Identification of lipids and lipid-binding proteins in phloem exudates from *Arabidopsis thaliana*. *Journal of Experimental Botany*, 63, 3603-3616.

Mousavi S.A.R., Chauvin A., Pascaud F., Kellenberger S. & Farmer E.E. (2013) *GLUTAMATE RECEPTOR-LIKE* genes mediate leaf-to-leaf wound signalling. *Nature*, 500, 422-429.

Xiao S., Gao W., Chen Q.F., Chan S.W., Zheng S.X., Ma J.Y., Wang M.F., Welti R. & Chye M.L. (2010) Overexpression of Arabidopsis acyl-CoA binding protein ACBP3 promotes starvation-induced and age-dependent leaf senescence. *Plant Cell*, 22, 1463-1482.
