# Supplementary material for: Depletion of Arabidopsis ACYL-COA-BINDING PROTEIN3 Affects Fatty Acid Composition in the Phloem
Source: Front Plant Sci. 2018 Jan 25;9:2. doi: 10.3389/fpls.2018.00002 (PMC5789640; doi:10.3389/fpls.2018.00002)
Supplement: Supplementary file 2 [file Supplementary_IEM_Images.PDF]

In this supplementary file, it provides all raw images that were used for the quantification of gold particle densities in companion cells (CC), sieve elements (SE) and extracellular space (ES) (Figure 2E).

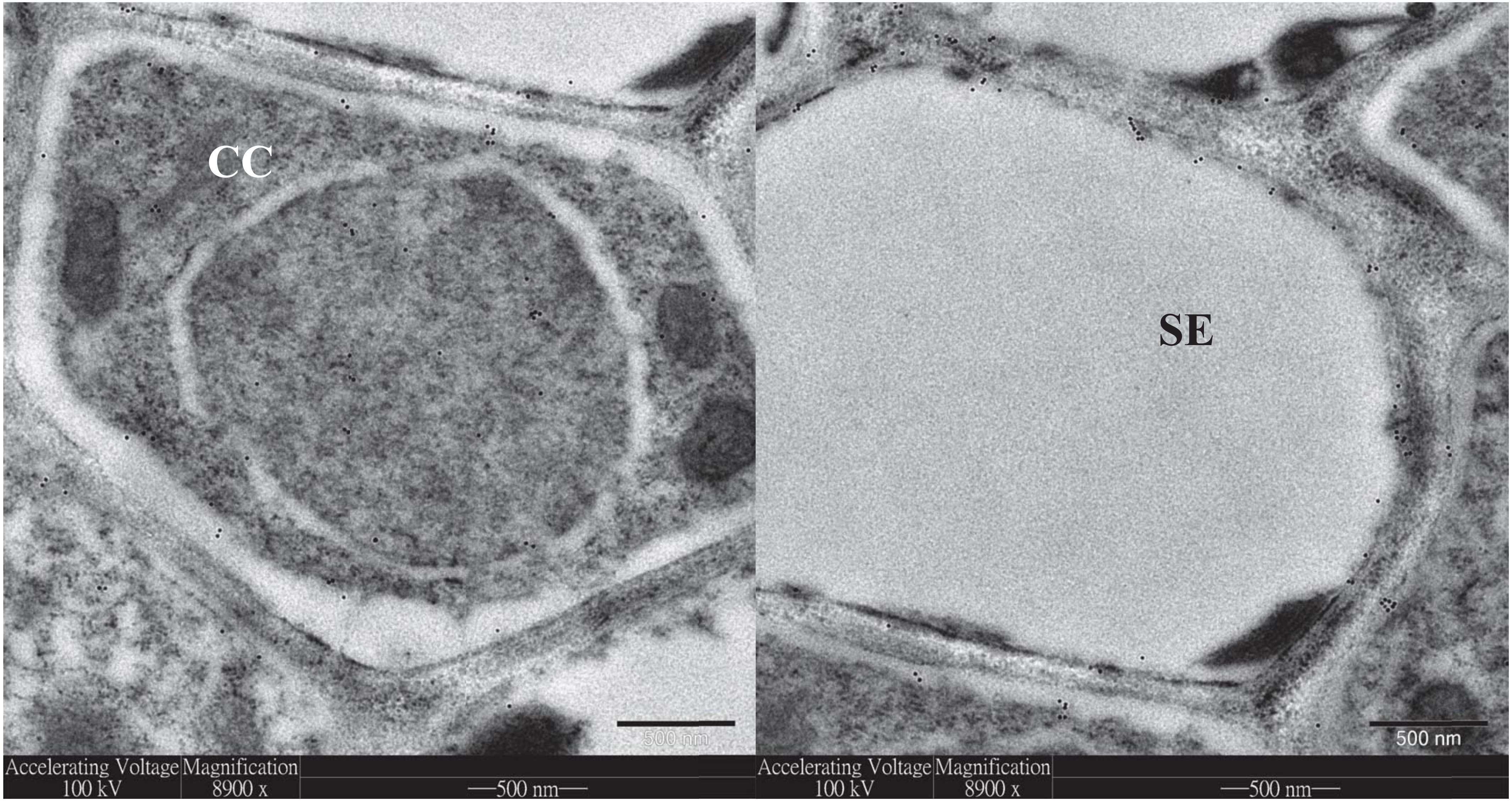
CC
500 nm
Accelerating Voltage
100 kV
Magnification
8900 x
500 nm
SE
500 nm
Accelerating Voltage
100 kV
Magnification
8900 x
500 nm

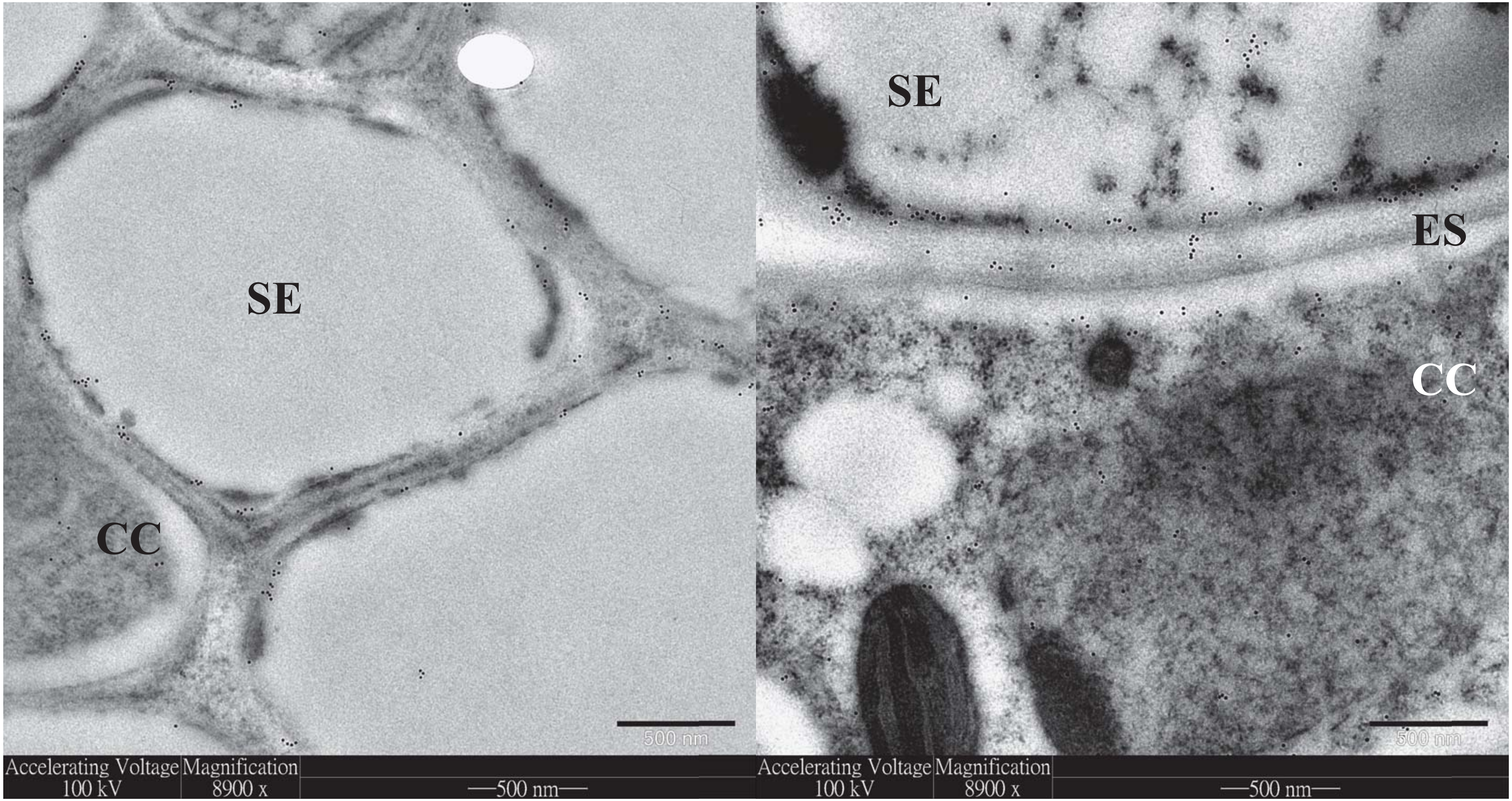
SE
CC
500 nm
Accelerating Voltage
100 kV
Magnification
8900 x
500 nm
SE
ES
CC
500 nm
Accelerating Voltage
100 kV
Magnification
8900 x
500 nm

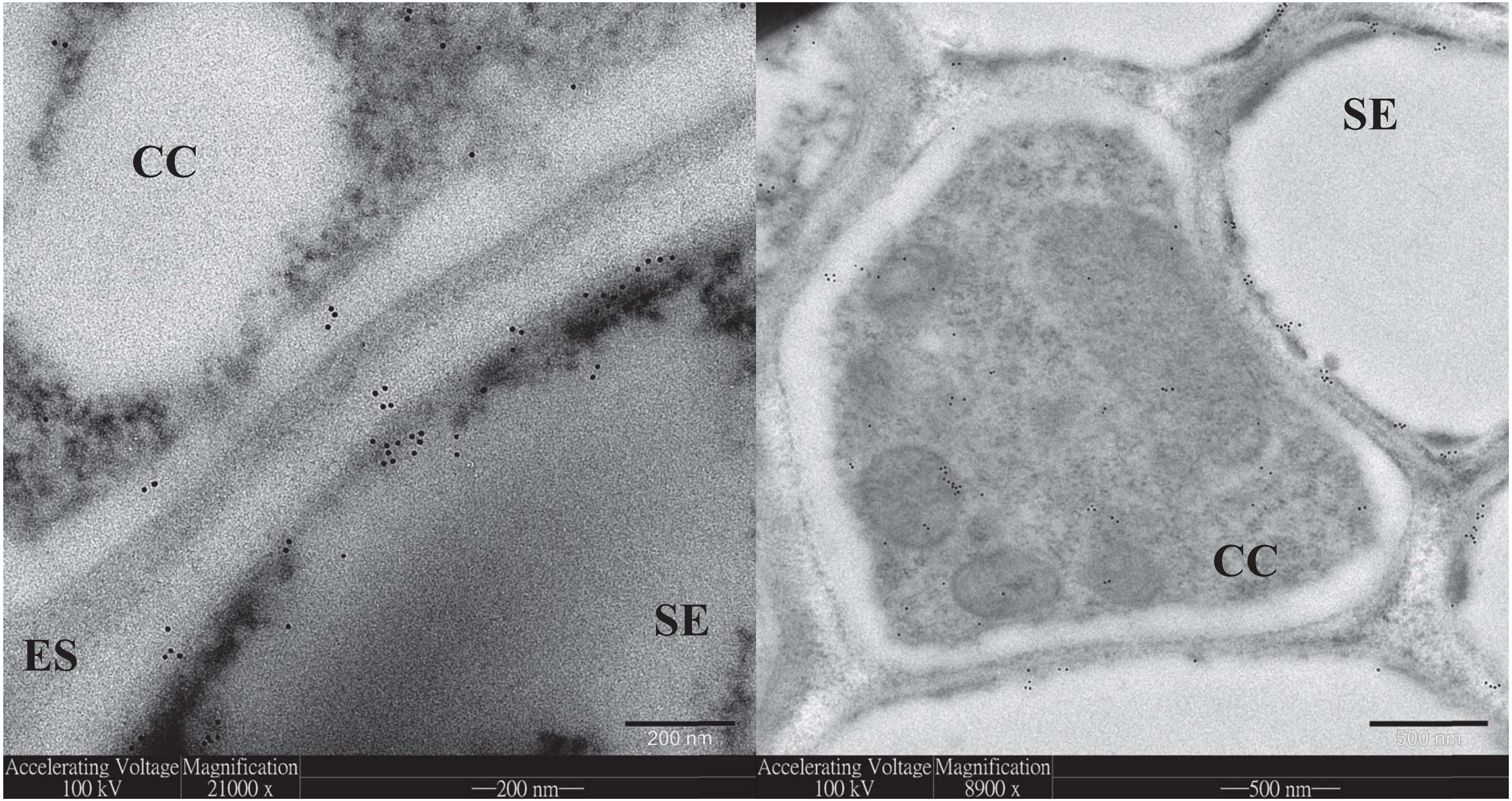
CC
ES
SE
200 nm
Accelerating Voltage
100 kV
Magnification
21000 x
—200 nm—
SE
CC
500 nm
Accelerating Voltage
100 kV
Magnification
8900 x
—500 nm—

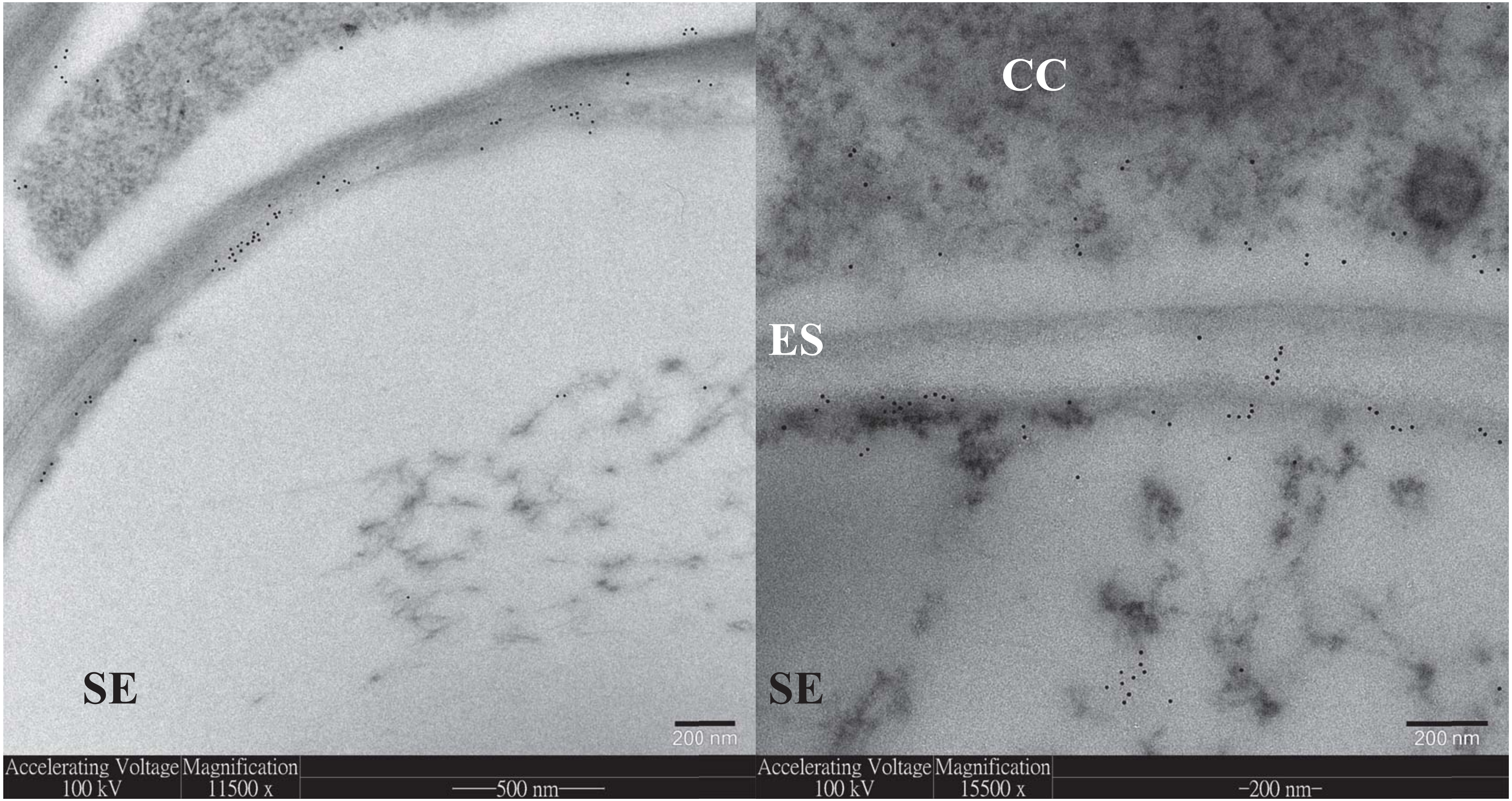
CC
ES
SE
SE
200 nm
200 nm
Accelerating Voltage
100 kV
Magnification
11500 x
500 nm
Accelerating Voltage
100 kV
Magnification
15500 x
200 nm

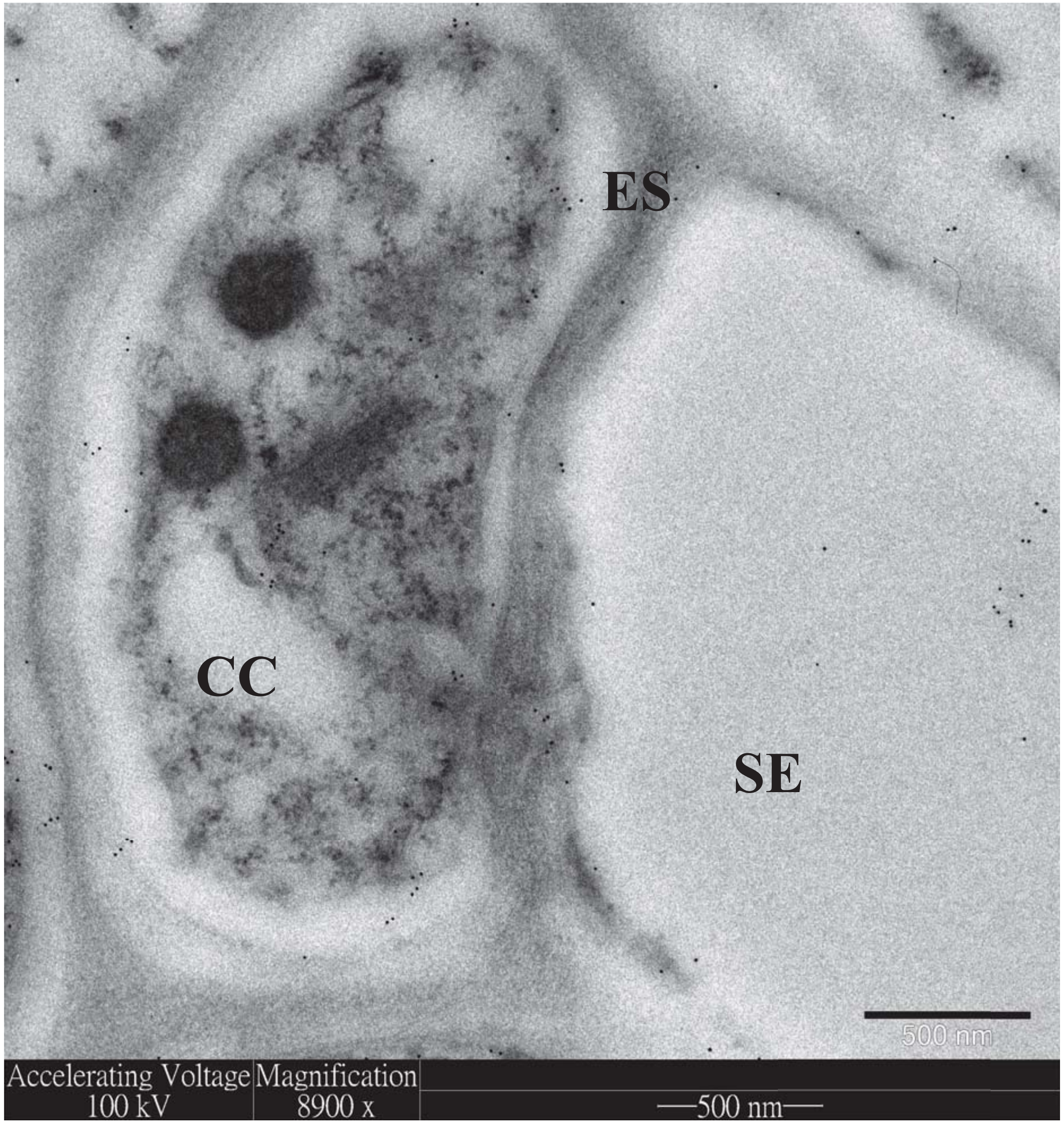
ES
CC
SE
500 nm
Accelerating Voltage
100 kV
Magnification
8900 x
500 nm
